# Tmem174, a regulator of phosphate transporter prevents hyperphosphatemia

**DOI:** 10.1038/s41598-022-10409-3

**Published:** 2022-04-15

**Authors:** Sumire Sasaki, Yuji Shiozaki, Ai Hanazaki, Megumi Koike, Kazuya Tanifuji, Minori Uga, Kota Kawahara, Ichiro Kaneko, Yasuharu Kawamoto, Pattama Wiriyasermkul, Tomoka Hasegawa, Norio Amizuka, Ken-ichi Miyamoto, Shushi Nagamori, Yoshikatsu Kanai, Hiroko Segawa

**Affiliations:** 1 Department of Applied Nutrition, Institute of Biomedical Sciences, Tokushima University Graduate School, Tokushima, Japan; 2 Department of Bio-System Pharmacology, Graduate School of Medicine, Osaka University, Osaka, Japan; 3 Department of Laboratory Medicine, The Jikei University School of Medicine, Tokyo, Japan; 4 Developmental Biology of Hard Tissue, Faculty of Dental Medicine, Hokkaido University, Sapporo, Japan; 5 Graduate School of Agriculture, Ryukoku University, Ohtsu, Japan

**Keywords:** Nephrology, Kidney diseases, Chronic kidney disease, Phosphorus metabolism disorders

## Abstract

Renal type II sodium-dependent inorganic phosphate (Pi) transporters NaPi2a and NaPi2c cooperate with other organs to strictly regulate the plasma Pi concentration. A high Pi load induces expression and secretion of the phosphaturic hormones parathyroid hormone (PTH) and fibroblast growth factor 23 (FGF23) that enhance urinary Pi excretion and prevent the onset of hyperphosphatemia. How FGF23 secretion from bone is increased by a high Pi load and the setpoint of the plasma Pi concentration, however, are unclear. Here, we investigated the role of Transmembrane protein 174 (Tmem174) and observed evidence for gene co-expression networks in NaPi2a and NaPi2c function. Tmem174 is localized in the renal proximal tubules and interacts with NaPi2a, but not NaPi2c. In Tmem174-knockout (KO) mice, the serum FGF23 concentration was markedly increased but increased Pi excretion and hypophosphatemia were not observed. In addition, Tmem174-KO mice exhibit reduced NaPi2a responsiveness to FGF23 and PTH administration. Furthermore, a dietary Pi load causes marked hyperphosphatemia and abnormal NaPi2a regulation in Tmem174-KO mice. Thus, Tmem174 is thought to be associated with FGF23 induction in bones and the regulation of NaPi2a to prevent an increase in the plasma Pi concentration due to a high Pi load and kidney injury.

## Introduction

NaPi2a and NaPi2c (SLC34A1/NPT2A/NaPi2a and SCL34A3/NPT2C/NaPi2c), sodium-dependent phosphate transporters responsible for inorganic phosphate (Pi) reabsorption in the kidney, are essential molecules for regulating the plasma Pi concentration. Both transporters are predominantly expressed at the apical side in the proximal tubules of the kidney^1,2^. Parathyroid hormone (PTH) and fibroblast growth factor 23 (FGF23) are the main contributing hormones regulating the renal NaPi2a and NaPi2c transporters^1–5^. In rodents, NaPi2a plays a central role in Pi reabsorption^6,7^. NaPi2a has a PDZ (PSD-95, Disc-large, ZO-1)-binding motif at its C-terminus and binds to Na^+^/H^+^ exchanger regulatory factor (NHERF)1 to form a complex at the apical membrane of proximal tubular cells^8–10^. PTH and FGF23 phosphorylate NHERF1, thereby dissociating the complex, and NaPi2a is endocytosed and degraded in lysosomes^1,11,12^. In this way, phosphaturic hormones reduce NaPi2a and enhance urinary Pi excretion.

Dietary Pi intake regulates urinary Pi excretion by altering plasma PTH and FGF23 levels^5^. Both phosphaturic hormones affect NaPi2a and NaPi2c expression in the proximal tubular cells^1,2,5^. With a low Pi diet intake, plasma PTH and FGF23 levels decrease, and the NaPi2a/NaPi2c levels in the proximal tubular cells increase. Therefore, urinary Pi excretion is reduced. In contrast, with a high dietary Pi load, plasma PTH and FGF23 levels are increased to promote the internalization of NaPi2a and NaPi2c in the proximal tubular cells. As a result, urinary Pi excretion is enhanced, and the onset of hyperphosphatemia is prevented. The mechanisms underlying both the induction of phosphaturic hormones by dietary Pi and regulation of the serum Pi concentration, however, remain unclear. Therefore, we searched for new mediators of NaPi2a/NaPi2c expression/trafficking.

Gene co-expression networks (GCNs) represent gene–gene interactions and while they do not contain information about regulation direction, they allow for the simultaneous analysis of many genes and their potential relationships with each other^13–16^. In the present study, we focused on transmembrane protein 174 (Tmem174), which is strongly correlated with slc34a1 in the GCNs. Tmem174 is localized in the renal proximal tubule apical membrane. The present study showed that Tmem174-knockout (KO) mice exhibit abnormal fluctuations in the plasma Pi levels in response to dietary Pi. Tmem174 binds to NaPi2a on the cell membrane and is considered to be involved in the regulation of NaPi2a by PTH and FGF23. The roles of Tmem174 in the control of plasma Pi are discussed.

## Results

### COXPRESdb search indicates co-expression of transmembrane protein 174 (Tmem174) with renal NaPi transporters

**Figure 1:**
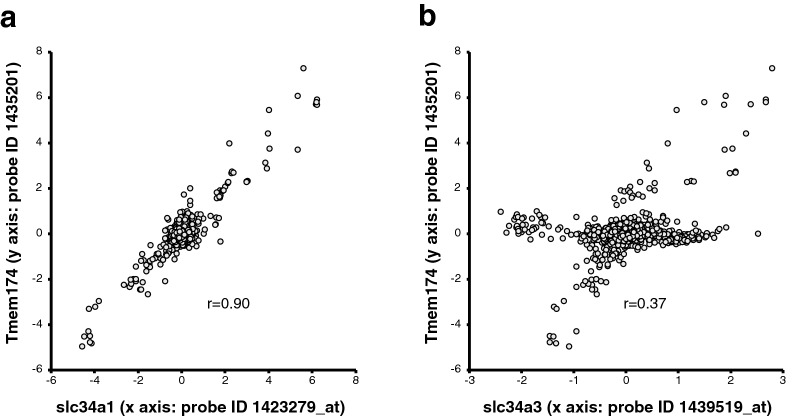
Genes co-expressed with renal slc34a NaPi transporters. The genes co-expressed with slc34a1 or slc34a3 were identified by database search on COEXPRESdb, as listed in Supplemental Tables S1 and S2. Correlation profile of gene expression between (**a**) slc34a1 (x axis: probe ID 1423279_at), or (**b**) slc34a3 (x axis: probe ID 1439519_at), and Tmem174 (y axis: probe ID 1,435,201).

To identify genes co-regulated with slc34a1 or slc34a3 mouse NaPi transporters, we searched for genes using the COXPRESdb v7^15^. The top 20 genes are listed in Supplemental Tables S1 and S2, and the transmembrane protein 174 (Tmem174) gene was identified as a significant gene co-expressed with slc34a1 and slc34A3. The correlation coefficient (r) for gene expression levels between slc34a1 or slc34a3 and Tmem174 was 0.90 and 0.37, respectively (Fig. 1a,b).

TMEM174 was originally identified among a large pool of genes by high-throughput cell screening technology to isolate functional genes and provide insight into the mechanisms of gene function^17–19^. The full-length amino acid sequences of Tmem174 in mouse (NP_080961.1), rat (NP_001019469.1), and human (NP_694949.1) are reported in the NCBI database. The putative Tmem174 protein comprises 243 amino acids with 2 transmembrane domains.

### Tissue localization of Tmem174 expression and possible involvement in Pi homeostasis

**Figure 2:**
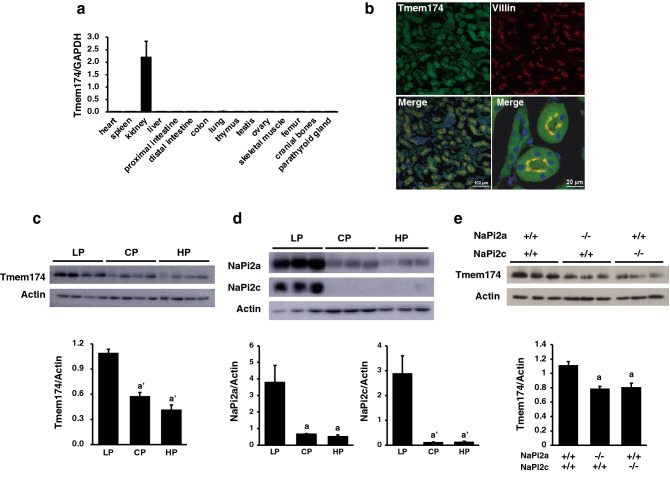
Tissue localization of Tmem174 expression and possible involvement in Pi homeostasis. (**a**) Tmem174 mRNA level in several tissues of wild-type (WT) mice by real-time PCR. Male mice at 8 weeks of age (n = 5–9) were used. Glyceraldehyde-3-phosphate dehydrogenase (GAPDH) was used as an internal control. Values are mean ± SE. (**b**) Immunofluorescence staining Tmem174 (green) in renal section of 8-week-old WT mice. DAPI (blue), Villin (red). Sections were prepared from mouse kidney embedded in OCT compound and frozen. (**c**,**d**) Western blotting analyses of the renal brush border membrane vesicles (BBMVs) isolated from the WT mice (n = 3–5) fed a low Pi (LP: 0.02%), control Pi (CP: 0.6%), and high Pi (HP: 1.2%) diet. Each lane was loaded with 20 μg of BBMVs. Actin was used as an internal control. Values are mean ± SE. ^a^*p* < 0.05, ^a’^*p* < 0.01 vs. LP. Experiments were performed in triplicate. (**e**) Western blotting analysis of the renal BBMVs isolated from the kidney of 8-week-old NaPi2a^+/+^ NaPi2c^+/+^, NaPi2a^−/−^, and NaPi2c^−/−^ mice (n = 3–5). Each lane was loaded with 20 μg of BBMVs. Actin was used as an internal control. Values are mean ± SE. ^a^*p* < 0.05 vs. NaPi2a^+/+^NaPi2c^+/+^ mice. Experiments were repeated at least 3 times.

Expression of Tmem174 mRNA was analyzed by real-time polymerase chain reaction (PCR) using mouse tissues. As reported in human tissue^17^, mouse Tmem174 mRNA was markedly higher in the kidney compared with other tissues (Fig. 2a). Tmem174 protein expression was detected at the apical membrane of renal proximal tubular cells, but not in the distal tubule (Fig. 2b). Next, we examined whether the renal Tmem174 protein expression was regulated by dietary Pi regulation and deletion of renal Pi transporters NaPi2a, or NaPi2c (Fig. 2c–e). A low Pi (LP) diet significantly induced renal Tmem174 protein expression compared with control Pi (CP) and high Pi (HP) diets, similar to the response of renal Pi transporters to dietary Pi content (Fig. 2c,d). Furthermore, deletion of renal NaPi transporters (NaPi2a-KO and NaPi2c-KO mice) significantly reduced the renal Tmem174 protein expression levels compared with NaPi2a^+/+^ NaPi2c^+/+^ mice (Fig. 2e).

### Characterization of Tmem174^−/−^ mice fed standard mouse chow

**Figure 3:**
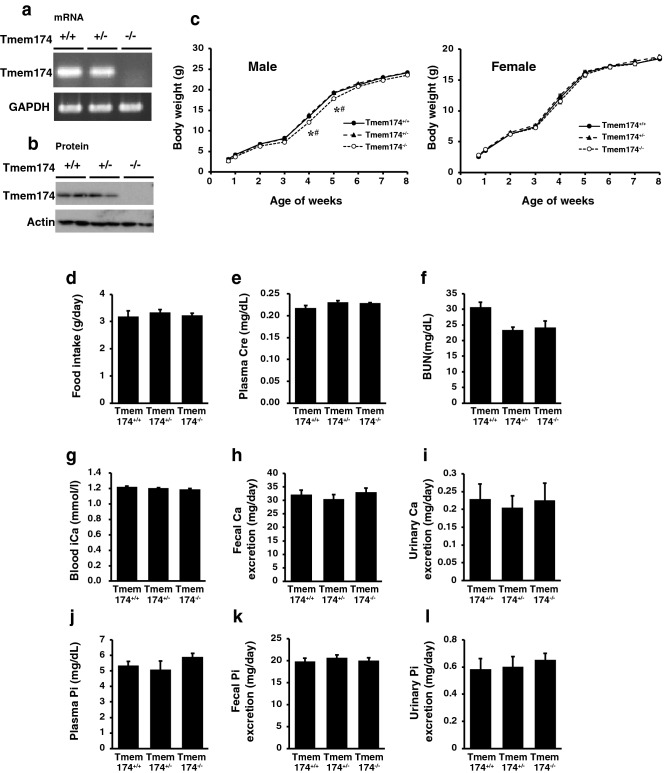
Characteristics of Tmem174^−/−^ mice. Expression of Tmem174 mRNA and protein in Tmem174^+/+^, Tmem174^+/-^, and Tmem174^−/−^ mice. PCR and Western blotting analysis in the kidney of mice. (**a**) Aliquots of each PCR product were electrophoresed on a 1.5% agarose gel. Glyceraldehyde-3-phosphate dehydrogenase (GAPDH) was used as the internal control. (**b**) Western blotting analysis of BBMVs isolated from the kidneys of Tmem174^+/+^, Tmem174^+/-^, and Tmem174^−/−^ mice. Each lane was loaded with 20 μg of BBMV. Actin was used as the internal control. (**c**) Growth curves for male and female Tmem174^+/+^, Tmem174^+/-^, and Tmem174^−/−^ mice. Values are mean ± SE (n = 10–30). *p < 0.05 vs. Tmem174^+/+^, ^#^*p* < 0.05 vs. Tmem174^+/-^ mice. Metabolic cages were used for measurement of 24-h food intake (g/day), and collection of urine, and feces from mice. (**d**) Food intake, (**e**) plasma creatinine, (**f**) plasma blood urea nitrogen, (**g**) blood ionized Ca, (**h**) fecal Ca excretion, (**i**) urinary Ca excretion, (**j**) plasma Pi, (**k**) fecal Pi excretion, (**l**) urinary Pi excretion. Male Tmem174^+/+^, Tmem174^+/-^, and Tmem174^−/−^ mice at 8–9 weeks of age (n = 30–50). Values are mean ± SE.

To generate Tmem174-null mice, we replaced the genomic region extending from Tmem174 exon 1 to the 5’ portion of exon 2 with a neomycin-resistant gene (Supplemental Fig. S1a). We confirmed the mutant genomic DNA isolated from transfected ES clones by Southern blot analysis, and the mice genotype by PCR analysis (Supplemental Fig. S1b,c). Reverse transcriptase-PCR with Tmem174-specific primers and Western blotting analysis confirmed the absence of detectable renal Tmem174 mRNA and protein expression in Tmem174^−/−^ mice (Fig. 3a,b). Male and female Tmem174^−/−^ mice showed similar weight gain compared with Tmem174^+/+^ and Tmem174^+/-^ mice (Fig. 3c). To measure food intake, and urine and fecal biochemical data, mice were individually placed in metabolic cages. Tmem174^−/−^ mice did not show a significant difference in food intake, plasma creatinine, plasma blood urea nitrogen (BUN), blood ionized Ca and plasma Pi concentrations, fecal and urinary Ca, Pi excretion levels, or other blood biochemistry parameters compared with Tmem174^+/+^ and Tmem174^−/−^ mice (Fig. 3d–l, and Supplemental Table S3).

### Trends in Pi-regulating hormones in Tmem174^−/−^ mice

**Figure 4:**
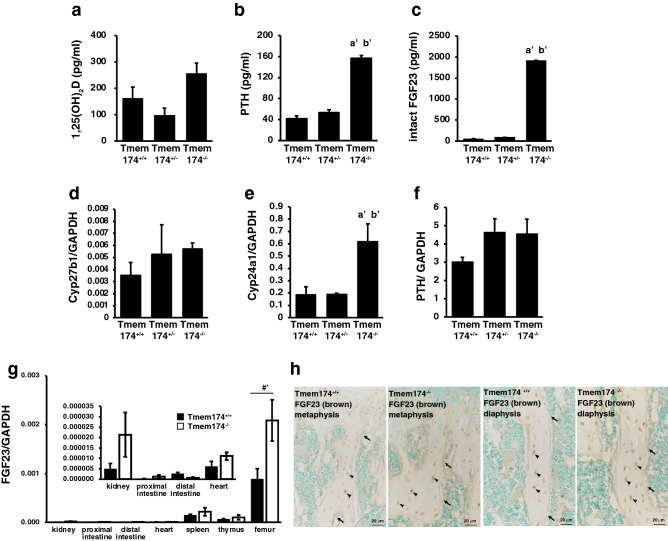
Effects of Deletion of Tmem174 on Pi homeostasis. (**a**–**c**) Plasma1,25(OH)_2_D, plasma intact PTH, serum intact FGF23 levels of Male Tmem174^+/+^, Tmem174^+/-^, and Tmem174^−/−^ mice at 8–9 weeks of age. Values are mean ± SE (n = 10–20). ^a’^*p* < 0.01 vs. Tmem174^+/+^ mice. ^b’^*p* < 0.01 vs. Tmem174^+/-^ mice. (**d**–**g**) Real-time PCR analysis. GAPDH was used as an internal control. Values are mean ± SE (n = 10–15). ^a’^*p* < 0.01 vs. Tmem174^+/+^ mice. ^b’^*p* < 0.01 vs. Tmem174^+/-^ mice. ^#’^*p* < 0.01. (**h**) Immunohistochemistry of FGF23 (brown color) in metaphyseal and diaphysis trabeculae bones of Tmem174^+/+^ and Tmem174^−/−^ male mice (8-week-old). Arrows: osteoblast/preosteoblast, arrowhead: osteocyte.

Plasma 1,25(OH)_2_D levels were not significantly different among the 3 groups (Fig. 4a). Plasma PTH, and especially serum intact FGF23 levels were markedly higher in Tmem174^−/−^ mice than in Tmem174^+/+^ and Tmem174^+/-^ mice (Fig. 4b,c). Renal 25-hydroxyvitamin D-1 alpha hydroxylase (Cyp27b1) mRNA levels were not significantly different among the 3 groups, but renal 25-hydroxyvitamin D-24-hydroxylase (Cyp24a1) mRNA levels were significantly higher in Tmem174^−/−^ mice than in Tmem174^+/+^ and Tmem174^+/-^ mice (Fig. 4d,e). Parathyroid PTH mRNA levels were not significantly different among the three groups (Fig. 4f). FGF23 mRNA was mainly detected in osseous-tissues but has been found in other tissues as well^20^. Expression of FGF23 mRNA in the bone was highly increased in Tmem174^−/−^ mice compared with Tmem174^+/+^ mice, and to a lower extent in the spleen and thymus (Fig. 4g). FGF23 mRNA levels in the kidney of Tmem174^−/−^ mice tended to be increased compared with that in control mice but were very low compared with that in the bone tissue. In both Tmem174^+/+^ and Tmem174^−/−^ mice, FGF23 immunostaining was observed in osteocytes and osteoblasts/preosteoblasts, with no difference in the localization patterns. The number of FGF23-positive cells, however, tended to be increased in Tmem174^−/−^ mice (Fig. 4h).

### Bone histochemical analysis in Tmem174^−/−^ mice

**Figure 5:**
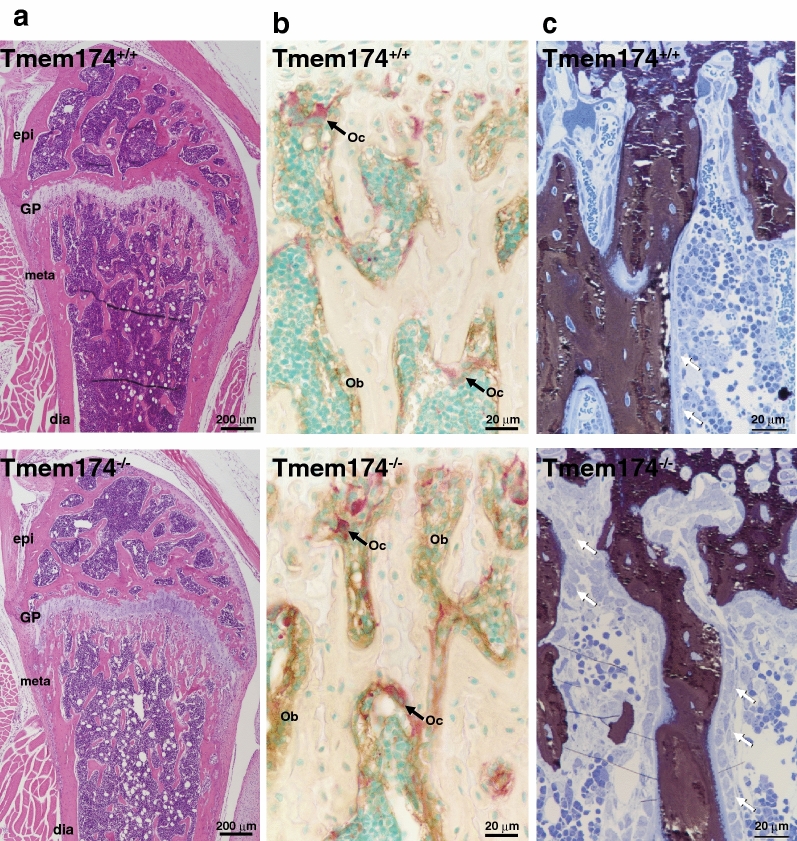
Bone analysis in Tmem174^−/−^ mice. Histological analysis of longitudinal femoral sections of 8-week-old Tmem174^+/+^ and Tmem174^−/−^ mice. (**a**) Hematoxylin/eosin staining, (**b**) Double staining of alkaline phosphatase (brown color) and tartrate-resistant acid phosphatase (red color), (**c**) von Kossa staining of metaphyseal trabeculae.

Bone analysis was performed in young (8-week-old) and aged (70-week-old) mice (Supplemental Fig. S2 and Fig. 5). Compared with Tmem174^+/+^ mice, neither 8-week-old nor 70-week-old Tmem174^−/−^ mice showed any abnormalities in the micro-computed tomography (micro-CT) analysis (Supplemental Fig. S2). Hematoxylin and eosin staining of the femurs from 8-week-old mice revealed that Tmem174^−/−^ mice had slightly more cancellous bone at the metaphysis compared with Tmem174^+/+^ mice (Fig. 5a). Consistently, bone histomorphometry showed that the BV/TV was greater in 8-week-old Tmem174^−/−^ mice than in Tmem174^+/+^ mice (Supplemental Fig. S2). There are no significant differences in Tb. Th, Ct. Th., or the width of growth plate between the Tmem174^+/+^ and Tmem174^−/−^ mice. In addition, alkaline phosphatase (ALP)/tartrate-resistant acid phosphatase staining revealed a tendency toward a thicker ALP-positive osteoblast/preosteoblast layer on the trabecular surface in Tmem174^−/−^ mice, and a similar number of tartrate-resistant acid phosphatase-positive osteoclasts between Tmem174^+/+^ and Tmem174^−/−^ mice, or a slightly higher number than in Tmem174^−/−^ mice (Fig. 5b).

Furthermore, the bone mineralization and osteoid layer thickness did not differ significantly between Tmem174^+/+^ and Tmem174^−/−^mice, whereas mature osteoblasts covered the bone surface in Tmem174^−/−^ mice (Fig. 5c).

### Renal NaPi transporter expression in Tmem174^−/−^ mice

**Figure 6:**
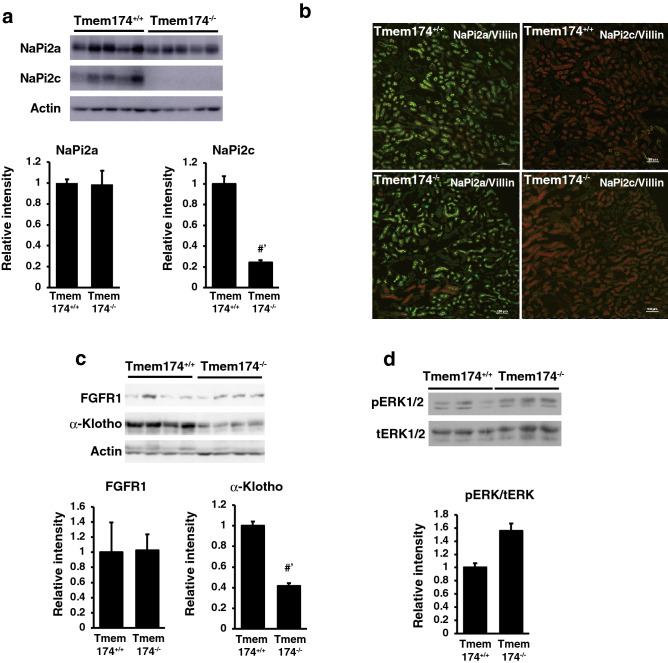
Deletion of Tmem174 and renal NaPi transporters. (**a**) Western blot analysis of NaPi transporters in Tmem174^+/+^ and Tmem174^−/−^ mice (8-week-old mice, n = 5, respectively). Each lane was loaded with 20 μg of BBMVs. Actin was used as an internal control. Values are mean ± SE. ^#’^*p* < 0.01. Experiments were repeated at least 3 times. (**b**) Immunofluorescence staining of NaPi2a or NaPi2c (green) in renal sections of 8-week-old Tmem174^+/+^ and Tmem174^−/−^ mice. DAPI (blue), Villin (red). Sections were prepared from kidneys embedded in the OCT compound and frozen. Scale bar; 100 μm. (**c**,**d**) Western blotting analysis of FGFR, α-Klotho (**c**), and total ERK1/2/phosphorylation ERK1/2 (**d**) levels. Each lane was loaded with 20 μg of cortical membranes (**c**) or whole homogenate (**d**). Actin was used as an internal control. Values are mean ± SE. ^#’^*p* < 0.01. Experiments were repeated at least 3 times.

Immunoblotting analysis using brush border membrane vesicles (BBMVs) and immunofluorescence staining showed the disappearance of only renal NaPi2c protein expression in Tmem174^−/−^ mice compared with Tmem174^+/+^ mice (Fig. 6a,b). In contrast, however, NaPi2a protein expression was not different between Temm174^+/+^ or Tmem174^−/−^ mice (Fig. 6a,b). Real time-PCR showed significantly decreased slc34a1 and slc34a3 mRNA levels in Tmem174^−/−^ mice compared with Tmem174^+/+^ mice (Supplemental Fig. S3a,b).

Next, we examined the levels of each type of phosphaturic hormone receptor in the kidney. The levels of renal FGF receptor 1 (FGFR1) and PTH receptor (PTHR) mRNA were the same between Tmem174^+/+^ and Tmem174^−/−^ mice, and renal FGFR4 and α-Klotho mRNA levels were significantly decreased in Tmem174^−/−^ mice compared with Tmem174^+/+^ mice (Supplemental Fig. S3c–f). FGFR1 protein expression was not different between Tmem174^+/+^ and Tmem174^−/−^ mice, and α-Klotho protein expression was significantly decreased in Tmem174^−/−^ mice compared with Tmem174^+/+^ mice (Fig. 6c). Phosphorylation of the extracellular signal-regulated kinase (ERK)1/2 also was not different between Tmem174^+/+^ and Tmem174^−/−^ mice (Fig. 6d).

### Scaffold protein levels in the renal proximal tubules of Tmem174 KO mice

**Figure 7:**
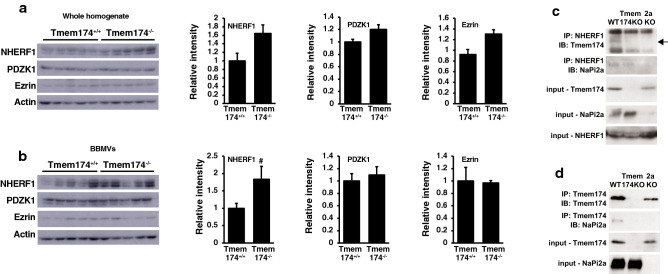
Scaffold protein levels in Tmem174^−/−^ mice. Western blot analysis of scaffold proteins in Tmem174^+/+^ and Tmem174^−/−^ mice fed normal mouse chow. Each lane was loaded with 20 μg of renal whole homogenate (**a**) and BBMVs (**b**). Actin was used as an internal control. Values are mean ± SE. ^#^*p* < 0.05. Immunoprecipitation of NHERF1 (**c**) or Tmem174 (**d**) from renal BBMV lysates of WT, Tmem174-KO and NaPi2a-KO mice fed the normal mouse chow and immunoblotting with anti-Tmem174, -NaPi2a or -NHERF1 antibodies. Arrow indicates the Tmem174. Experiments were repeated at least 3 times.

NaPi2a trafficked to the apical membrane is dependent on its association with PDZ-containing proteins^1,8,9,21–23^. PTH and FGF23 regulate Pi excretion by controlling the NaPi2a/NHERF1 association^1,10^. Immunoblot analysis using renal BBMVs and whole homogenate showed that NHERF1 protein expression was significantly or tended to be higher in Tmem174^−/−^ mice than in Tmem174^+/+^ mice (Fig. 7a,b).

Next, we examined the interaction between Tmem174 and NaPi2a/NHERF1 using renal BBMVs of wild-type (WT), Tmem174-KO, and NaPi2a-KO mice (Fig. 7c,b). NHERF1 immunoprecipitation analysis revealed a NaPi2a/NHERF1 interaction in both WT and Tmem174-KO mice. In contrast, NHERF1 immunoprecipitation analysis detected Tmem174 protein in WT mice, but not in NaPi2a-KO mice (Fig. 7c). These findings suggest that Tmem174 binds to NHERF1 in the presence of NaPi2a but cannot interact in the absence of NaPi2a. Tmem174 immunoprecipitation analyses showed an interaction between Tmem174 and NaPi2a (Fig. 7d).

### Response to the dietary Pi content in Tmem174 KO mice

**Figure 8:**
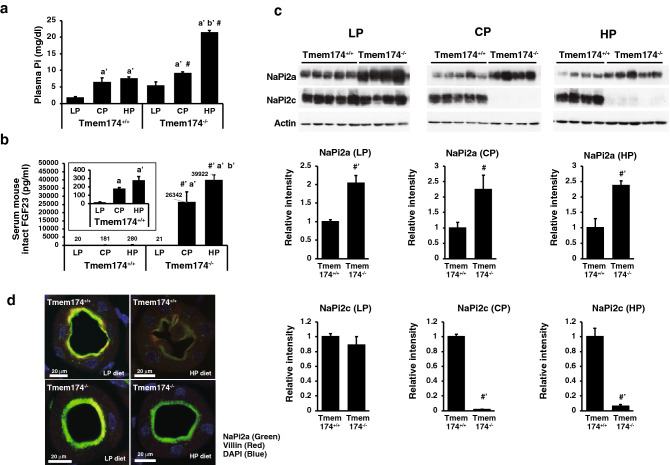
Dietary Pi regulation in Tmem174^−/−^ mice. Male Tmem174^+/+^ and Tmem174^−/−^ mice (n = 5) fed a low Pi (LP: 0.02%), control Pi (CP: 0.6%), or high Pi (HP: 1.2%) diet for 12 days. (**a**) Plasma Pi, (**b**) Serum intact FGF23, Values are mean ± SE. ^#^*p* < 0.05, ^#’^*p* < 0.01 vs same diet, ^a^*p* < 0.05, ^a’^*p* < 0.01 vs LP, ^b’^*p* < 0.01 vs. CP of same genotype. (**c**) Western blotting analyses. Each lane was loaded with 20 μg of BBMVs. Actin was used as an internal control. Values are mean ± SE. ^#^*p* < 0.05, ^#’^*p* < 0.01. (**d**) Immunostaining of renal NaPi2a (Green) localization in the kidney sections of mice fed the LP (0.02% Pi) and HP (1.2% Pi) diet for 7 days. DAPI (Blue), Villin (Red). Experiments were performed in triplicate.

We examined fluctuations in the plasma Pi levels due to differences in the dietary Pi content (Fig. 8a). Tmem174^−/−^ mice fed the HP diet had markedly higher plasma Pi levels than Tmem174^+/+^ mice. Furthermore, Tmem174^−/−^ mice fed the CP or HP diets had extremely high serum FGF23 levels compared with Tmem174^+/+^ mice fed an equivalent diet (Fig. 8b). In addition, renal NaPi2a, but not NaPi2c, protein expression levels were significantly higher in Tmem174^−/−^ mice fed the LP, CP, or HP diet compared with Tmem174^+/+^ mice fed the same diet (Fig. 8c). In contrast, the CP diet and HP diet significantly suppressed renal NaPi2c protein expression levels in Tmem174^−/−^ mice compared with Tmem174^+/+^ mice (Fig. 8c). NaPi2a immunofluorescence staining was strongly detected at the apical membrane of proximal tubular cells both in Tmem174^+/+^ and Tmem174^−/−^ mice fed the LP diet (Fig. 8d). The HP diet suppressed NaPi2a staining at the apical membrane in Tmem174^+/+^ mice. In contrast, Tmem174^−/−^ mice fed the HP diet maintained NaPi2a strong immunostaining at the apical membrane of the proximal tubular cells (Fig. 8d).

### Response to FGF23 in Tmem174 KO mice

**Figure 9:**
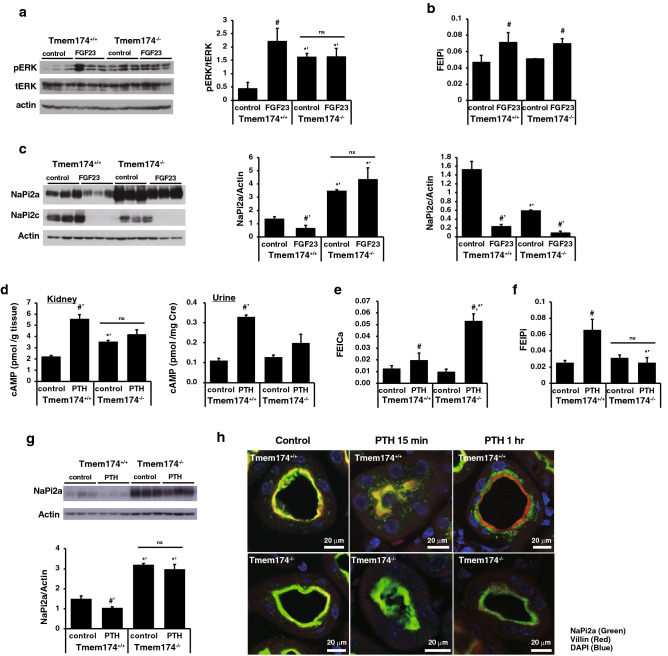
Abnormal regulation of phosphaturic action to renal NaPi2a Pi transporter in Tmem174^−/−^ mice. Tmem174^+/+^ and Tmem174^−/−^ mice (9–10-week-old mice, n = 3–5, respectively) were fed a 0.02% low Pi diet for 7 days to reduce endogenous FGF23. Exogenous FGF23 expression was performed using the Naked-DNA method, as described previously^26^. (**a**) Western blotting analysis of total ERK1/2 and phosphorylation ERK1/2 in Tmem174^+/+^ and Tmem174^−/−^ mice. Each lane was loaded with 20 μg of the whole homogenate. Values are mean ± SE. ^#^*p* < 0.05 vs control of the same genotype, *’*p* < 0.01 vs same treatment of Tmem174^+/+^ mice. *ns* not significant. (**b**) FEIPi. Values are mean ± SE. ^#^*p* < 0.05 vs control of the same genotype. (**c**) Western blot analysis of NaPi transporters in Tmem174^+/+^ and Tmem174^−/−^ mice. Each lane was loaded with 20 μg of BBMVs. Actin was used as an internal control. *’*p* < 0.01 vs same treatment of Tmem174^+/+^ mice. Values are mean ± SE. ^#’^*p* < 0.01 vs control of the same genotype. *ns* not significant. Experiments were performed in triplicate. Tmem174^+/+^ and Tmem174^−/−^ mice (9–10-week-old mice, n = 3–5, respectively) were fed a 0.1% low Pi diet for 7 days to reduce endogenous PTH. Bovine PTH (1–34) was administered to Tmem174^+/+^ and Tmem174^−/−^ mice. Samples were collected after 15 min or 1 h administration of PTH. (**d**) cAMP level of the kidney and Urine of mice after 15 min PTH administration. (**e**) FEICa, (**f**) FEIPi. Samples were collected after 1 h administration of PTH. Values are mean ± SE. ^#^*p* < 0.05 vs control of the same genotype. *’*p* < 0.01 vs same treatment of Tmem174^+/+^ mice. ns; not significant. (**g**) Western blot analysis of NaPi2a in Tmem174^+/+^ and Tmem174^−/−^ mice. Each lane was loaded with 20 μg of BBMVs. Actin was used as an internal control. Values are mean ± SE. ^#’^*p* < 0.01 vs control of the same genotype. *’*p* < 0.01 vs same treatment of Tmem174^+/+^ mice. *ns* not significant. (**h**) Immunostaining of renal NaPi2a (Green) localization in the kidney sections of mice 15 min and 1 h after administration of PTH. DAPI (Blue). Villin (Red). Experiments were performed in triplicate.

Next, to confirm the effect of the phosphaturic action of FGF23 in Tmem174^−/−^ mice, mice were fed the LP diet to reduce endogenous FGF23. Exogenous FGF23 was expressed using the Naked-DNA method, as described previously^24–26^. As shown in Supplemental Fig. S4a, the LP diet significantly suppressed serum FGF23 levels in both Tmem174^+/+^ and Tmem174^−/−^ mice. We confirmed the exogenous FGF23 mRNA (hFGF23) expression in the liver of the FGF23 groups in both Tmem174^+/+^ and Tmem174^−/−^ mice at 4 days after Naked-DNA injection (Supplemental Fig. S4b)^24–26^. In Tmem174^+/+^ mice, but not in Tmem174^−/−^ mice, FGF23 increased the level of ERK1/2 phosphorylation compared with the control group (Fig. 9a). Interestingly, the ERK phosphorylation level was higher in the control Tmem174^−/−^ mice compared with the control Tmem174^+/+^ mice. FGF23 groups of both Tmem174^+/+^ and Tmem174^−/−^ mice showed significantly lower levels of renal α-Klotho protein expression compared with their control groups (Supplemental Fig. S4c). Renal Cyp27b1 mRNA levels were significantly suppressed, and Cyp24a1 mRNA levels were significantly increased in the FGF23 groups of both Tmem174^+/+^ and Tmem174^−/−^ mice compared with their control groups (Supplemental Fig. S4d,e). Slc34a1, but not slc34a3, mRNA levels were significantly suppressed in FGF23 groups of both Tmem174^+/+^ and Tmem174^−/−^ mice compared with the control group (Supplemental Fig. S4f,g). Urinary Pi excretion levels were slightly but significantly increased in FGF23 groups of both Tmem174^+/+^ and Tmem174^−/−^ mice compared with the control group (Fig. 9b). In Tmem174^+/+^ mice, both renal NaPi2a and NaPi2c protein levels were significantly suppressed after FGF23 Naked DNA injection compared with the control group (Fig. 9c). In Tmem174^−/−^ mice, FGF23 significantly suppressed only NaPi2c protein expression and not NaPi2a protein expression levels (Fig. 9c).

### Response to PTH in Tmem174 KO mice

Next, we investigated the effect of PTH on NaPi2a protein expression in Tmem174^+/+^ and Tmem174^−/−^ mice. The LP diet significantly suppressed plasma PTH levels in Tmem174^+/+^ and Tmem174^−/−^ mice, and the plasma PTH levels did not differ significantly between Tmem174^+/+^ and Tmem174^−/−^ mice fed the LP diet (Supplemental Fig. S4h). To confirm that there was no difference in PTH signaling, we measured the renal and urinary cAMP levels 15 min after administration of PTH. PTH significantly induced cAMP in the kidney of Tmem174^+/+^ mice, but not Tmem174^−/−^ mice (Fig. 9d). Like FGF23, renal cAMP levels were slightly, but significantly, higher in the control Tmem174^−/−^ mice than in the control Tmem174^+/+^ mice. Urinary cAMP was significantly increased or tended to increase with PTH in both Tmem174^+/+^ mice and Tmem174^−/−^ mice (Fig. 9d). Urinary Ca excretion was significantly increased 1 h after PTH administration in both Tmem174^+/+^ and Tmem174^−/−^ mice (Fig. 9e). Urinary Pi excretion, however, was significantly increased 1 h after PTH administration in Tmem174^+/+^ mice, but not in Tmem174^−/−^ mice (Fig. 9f). Furthermore, PTH significantly suppressed renal NaPi2a protein expression in Tmem174^+/+^ mice, but not in Tmem174^−/−^ mice (Fig. 9g). We confirmed NaPi2a internalization in the kidney of Tmem174^+/+^ mice administered PTH at 15 min and 1 h, as described previously (Fig. 9h, upper)^27^. In Tmem174^−/−^ mice, NaPi2a remained localized at the apical membrane after administration of PTH (Fig. 9h, bottom).

### Renal injury in Tmem174 KO mice

**Figure 10:**
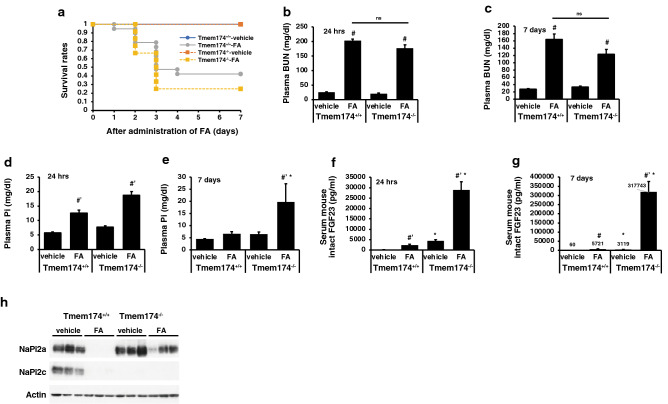
Renal injury in Tmem174^−/−^ mice. Male Tmem174^+/+^ and Tmem174^−/−^ mice (n = 10–30) were administered folic acid (FA; 240 μg/kg BW). (**a**) Survival curve, Plasma BUN at 24 h (**b**) and 7 days (**c**), plasma Pi at 24 h (**d**) and 7 days (**e**), and serum intact FGF23 at 24 h (**f**) and 7 days (**g**) after administration of FA. Values are mean ± SE. ^#^*p* < 0.05, ^#’^< 0.01 vs vehicle of the same genotype, *p < 0.05 vs same treatment of Tmem174^+/+^ mice. *ns* not significant. (**h**) Western blotting analysis of NaPi2a. Each lane was loaded with 20 μg of renal BBMVs of mice 24 h after administration of FA. Actin was used as an internal control.

Finally, we investigated the plasma Pi and BUN concentrations in a folic acid (FA)-induced acute kidney injury (AKI) model for 7 days. As shown in Fig. 10a, the deletion of Tmem174 shortened the lifespan of the AKI model mice. There were no significant differences in BUN levels at 24 h and 7 days after administration of FA between Tmem174^+/+^ and Tmem174^−/−^ mice (Fig. 10b,c). AKI-Tmem174^−/−^ mice, however, had significantly higher levels of plasma Pi at 24 h after FA administration compared with AKI- Tmem174^+/+^ mice, and the hyperphosphatemia was maintained only in Tmem174^−/−^ mice until 7 days after FA treatment (Fig. 10d,e). Furthermore, serum intact FGF23 levels were markedly higher in Tmem174^−/−^ mice at 24 h after FA treatment compared with Tmem174^+/+^ mice, and the markedly high levels of serum FGF23 were maintained only in Tmem174^−/−^ mice until 7 days after FA treatment (Fig. 10f,g). Renal NaPi2a protein levels were significantly reduced in Tmem174^+/+^ mice by FA administration, but only slightly reduced in Tmem174^−/−^ mice (Fig. 10h).

## Discussion

In the present study, we investigated the roles of a strongly correlated molecule (Tmem174) in the GCNs and Pi metabolism. Many transmembrane proteins grouped in the TMEM family are poorly described and have mostly unknown functions. After further characterization, they are generally renamed and reclassified into more specific categories such as GPCR proteins or ion channels^28,29^.

The Tmem174 protein was extremely highly expressed in the kidney and localized at the apical membrane of the renal proximal tubules. Dietary Pi content regulates the renal Tmem174 protein levels the same as NaPi2a. Immunoprecipitation experiments suggest that Tmem174 interacts with the NaPi2a/NHERF1 complex. Tmem174^−/−^ mice showed markedly increased serum FGF23 levels and significantly increased plasma PTH levels. Interestingly, renal NaPi2a protein levels were not decreased despite the marked increase in serum FGF23 levels. Based on studies of dietary Pi responses, the marked increase in plasma Pi levels in Tmem174^−/−^ mice are due to high Pi loading and may result from a resistance to NaPi2a regulation by PTH and FGF23. Thus, an abnormal dietary Pi response in Tmem174^−/−^ mice leads to dysregulation of plasma Pi levels.

Tmem174^−/−^ mice are characterized by (1) abnormally high serum FGF23 levels and (2) dietary Pi response abnormalities in the regulation of the plasma Pi concentration. The present findings suggest that Tmem174 binds to NaPi2a on the cell membrane and is involved in the internalization of NaPi2a. In Tmem174^−/−^ mice, no decrease in NaPi2a was observed despite high FGF23 and PTH concentrations. On the other hand, NaPi2c was significantly decreased. In addition, NHERF1 was significantly elevated in Tmem174^−/−^ mice. For these reasons, Tmem174 is considered to be a component of the NaPi2a/NHERF1 complex that receives signals from PTH and FGF23.

Although Tmem174 deficiency affects the NaPi2a/NHERF1 system and vitamin D-metabolizing enzymes, NaPi2c regulation are considered normal. PTH and FGF23 downregulate the NaPi2a/NHERF1 binary complex by activating 2 distinct signaling pathways that converge at NHERF1^1^. The internalization and degradation of NaPi2a increase Pi excretion and depend on activation of the ERK1/2 and serum/glucocorticoid- regulated kinase-1 pathways, resulting in phosphorylation of NHERF1^30^. PTH signals activate protein kinases A and C^12,31,32^. Triggered by phosphorylation of NHERF1, NaPi2a dissociates from NHERF1 and is then internalized^12^. Not all signals from the receptor have been examined in detail, but some signals were activated. In addition, Tmem174^−/−^ mice show an increase in renal cyp24a1 mRNA levels resulting from abnormally high serum FGF23 levels, but normal plasma 1,25(OH)_2_D levels. It is not clear why the plasma 1.25(OH)_2_D levels are not decreased. Certainly, suppression of cyp27b1 mRNA has not been observed, but because cyp24a1, which is involved in the degradation of 1,25 (OH)_2_D, is increased, it is assumed that plasma 1,25 (OH) _2_D levels would decrease. As expected, Tmem174 deficiency may affect the function of cyp24a1 (or cyp27b1) and the cellular metabolism of 1,25(OH)_2_D. As a member of the Tmem family, it is possible that it affects vitamin D-metabolizing enzymes as a constituent protein of intracellular organelles. Further studies on the role of Tmem174 in active vitamin D metabolism are needed.

In Tmem174^−/−^mice, NaPi2c protein was significantly reduced as compared with NaPi2a protein. The interaction of NaPi2c with NHERF3 (PDZK1) is more important than that with NHERF1^33,34^. In fact, NaPi2c expression is suppressed in NHERF3-KO mice^34^. We previously reported differences in signals between the phosphaturic action of FGF23 and the inhibitory effect on vitamin D synthesis^35^. Therefore, it is considered that the effect of Tmem174 deficiency is limited to the control function of NaPi2a. More detailed studies on the role of Tmem174 in NaPi2a regulation, such as the effect of NHERF1 on phosphorylation, are needed.

Another feature of Tmem174^−/−^ mice is enhanced FGF23 induction from the bone. High serum FGF23 levels cause the pathology observed in a mouse model of X-linked hypophosphatemia rickets (Hyp mice)^36,37^. On the other hand, Tmem174^−/−^ mice did not exhibit the abnormal bone morphology seen in Hyp mice and we speculate that this is because Tmem174^−/−^ mice do not develop hypophosphatemia. The bone analysis data suggest that a high PTH concentration affects fluctuations in the numbers of osteoblasts and osteoclasts. More detailed studies will help to clarify the role of Tmem174 in bone.

High FGF23 induction in Tmem174^−/−^ mice is improved by a low Pi diet. Therefore, renal Tmem174 is expected to signal dietary Pi levels to bone FGF23. On the other hand, in an FA-induced renal disorder model, a further increase in serum FGF23 concentration was observed in Tmem174^−/−^ mice. FGF23 induction is known to be independent of dietary Pi signals in an FA-acute kidney injury model^38^. Therefore, the increase in FGF23 in Tmem174^−/−^ mice may be independent of the signal of renal damage. The relationship between α-Klotho and Tmem174 as a mediator from the kidney remains unclear. α-Klotho plays an important role in phosphate regulation by　FGF23 as a co-receptor for FGFR1 in the kidney^39,40^. In Tmem174^−/−^ mice, renal α-Klotho levels are reduced by approximately 50%. Previous studies reported that a decrease in α-Klotho in the kidney triggers the induction of FGF23 from the bone^39,40^. In contrast, we speculate that the cause of the α-Klotho decrease in the Tmem174^−/−^ mouse kidney is the high concentration of serum FGF23. There are many possible causes for the decrease in klotho, but it is unclear from this study.

**Figure 11:**
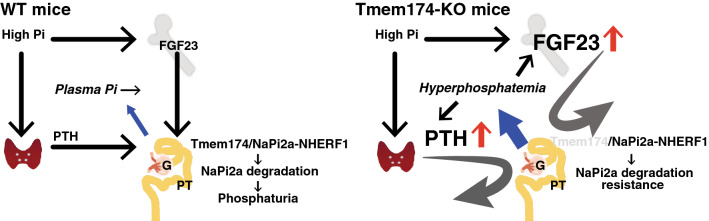
The putative role of Tmem174 in the control of plasma Pi levels. Tmem174 interacts with NaPi2a and is involved in internalization by PTH and FGF23. Under a high Pi load, urinary Pi excretion is enhanced by the internalization of NaPi2a induced by PTH and FGF23, and hyperphosphatemia is prevented. In Tmem174-KO mice, internalization of NaPi2a is resistant to PTH and FGF23. As a result, Pi reabsorption is maintained, and plasma Pi concentration increases. Tmem174 is expected to be a molecule that is associated with both the induction of phosphaturic hormones (PTH, FGF23) and the regulation of NaPi2a in the proximal tubules. *G* glomerulus, *PT* proximal tubule.

Finally, phosphaturic hormone is secreted in response to an excessive Pi load and acts on the kidneys to promote Pi excretion. The NaPi2a/NHERF1 complex has an important role. Tmem174 is expected to regulate the amount of NaPi2a in response to a Pi deficiency or excess and regulates the responsiveness of phosphaturic hormone. For example, vitamin D treatment in Hyp mice restores serum Pi levels by causing FGF23 resistance to NaPi2a/NHERF1^35,41^. Thus, Tmem174, a strongly correlated molecule with NaPi2a in the GCNs, is thought to be involved in the regulation of NaPi2a by PTH and FGF23 in the kidney and the prevention of hyperphosphatemia in response to a high dietary Pi load (Fig. 11).

## Materials and methods

### Experimental animals

All experimental procedures involving animals were conducted in accordance with the Tokushima University School of Medicine and Osaka University Graduate School of Medicine guidelines. This study was also carried out in compliance with the ARRIVE guidelines. All procedures involving the use of animals were subjected to approval from Tokushima University School of Medicine (T2019-126) and Osaka University Graduate School of Medicine (19-064-02) ethics committee.

Male and female C57B6/J mice were purchased from Charles River Laboratories Japan (Yokohama, Japan). NaPi2a-KO and NaPi2c-KO mice were maintained as described previously^7,42^. Mice were weaned at 4 weeks of age and provided free access to water and standard mouse chow containing 0.8% phosphorus (Oriental MF; ORIENTAL YEAST CO., LTD, Osaka, Japan).

### Generation of Tmem174-KO mice

Tmem174-deficient mice were generated by gene targeting. A targeting vector was constructed by replacing the 129 genomic Tmem174 loci (exon1 through part of exon2) with neo^r^ (Supplemental Fig. S1a). The targeting vector was introduced into 129 days 14 embryonic stem (ES) cells by electroporation, and clones that underwent homologous recombination were confirmed by Southern blot analysis (Supplemental Fig. S1b). Genomic DNA was extracted from tail clippings and amplified by PCR using specific primers (Supplemental Fig. S1c, Table 1).

**Table 1:** Primers for genotyping PCR.

Primer name	Sequences (5'–3')
Primer 1	ACATTCATCCTGATCGCTGTGTG
Primer 2	CGTGCAATCCATCTTGTTCAAT
Primer 3	GGAATTTAACCAGGGCAGCTTAA

### Dietary adaptation

For the dietary adaptation study, mice were fed a test diet based on modified AIN93G (low Pi; 0.02 or 0.1% Pi, control Pi; 0.6% Pi, and high Pi; 1.2% Pi), as described previously^43–45^.

### Effect of FGF23 and PTH on the regulation of NaPi transporter degradation

For exogenous FGF23 expression, TransIT-EE Hydrodynamic Delivery Solution was used as the TransIT in vivo gene delivery system (TaKaRa Bio, Shiga, Japan) as described previously^25,26^. For PTH injection, mice were administered bovine PTH (amino acid 1–34; Sigma-Aldrich, St. Louis, MO, USA) at a dose of 7.5 μg/100 g body weight, as described previously^46,47^.

### Metabolic cages to collect urine and fecal samples

Mice were individually placed in the metabolic cages at AM.10:00 for quantitative urine and fecal collection for 24 h with free access to food and water. Fecal samples were ashed according to a modified protocol, as described previously^35,43,48,49^.

### Biochemical measurements

Concentrations of Pi, Ca, and creatinine and BUN were determined using commercial kits (WAKO, Osaka, Japan). The fractional excretion index for Ca (FEICa) and Pi (FEIPi) was calculated as follows: urinary Ca or Pi/ (urinary creatinine /serum Ca or Pi). Concentrations of serum FGF23, plasma PTH, and 1,25(OH)_2_D were determined using the FGF23 ELISA kit (KAINOS Laboratories, Tokyo, Japan), intact PTH ELISA kit (Immunotopics Inc., San Clemente, CA, USA), and1,25-(OH)_2_ Vitamin D ELISA Kit (Immundiagnostik, Bensheim, Germany), respectively. Other blood clinical parameters were analyzed by automated methods. The renal and urinary cAMP levels were measured using Cyclic AMP Select EIA Kit (Cayman Chemical, Ann Arbor, MI, USA). Kidneys were flash-frozen in liquid nitrogen and dropped into 5–10 volumes of 5% trichloroacetic acid (TCA) solution. The kidneys were homogenized on ice, and the supernatant was transferred to a clean tube after removing the precipitate by centrifugation at 1500×*g* for 10 min. TCA was extracted from the samples using water-saturated ether. The samples were further heated at 70 °C for 5 min to remove any residual ether in the aqueous layer. The supernatant of the kidney extract was used for cAMP measurement according to the manufacturer’s instructions.

### RNA extraction, cDNA synthesis, and quantitative PCR

Mouse tissues were sampled, immediately submerged in RNAlater (Sigma-Aldrich), and stored at − 20 °C until use. Total cellular RNA from the sampled tissues was extracted and purified using ISOGEN (Wako, Osaka Japan) according to the manufacturer’s instructions. Complementary DNA (cDNA) was synthesized as described previously^35,43,48,49^. The template DNA was omitted for the negative control (-) for all PCR experiments. The PCR reaction was examined without reverse transcriptase (data not shown). Quantitative PCR was performed using ABI PRISM 7500 (Applied Biosystems, Foster City, CA) as described previously^35,43,45,48,49^. The reaction mixture consisted of 10 μl of SYBR Premix Ex Taq, ROX Reference Dye II (Perfect Real Time, TaKaRa Bio) with specific primers (Table 2).

**Table 2:** Primers for RT-PCR.

Primer	Sense (Sequences; 5'–3')	Antisense (Sequences; 5'–3')
Cyp24A1	TGGGAAGATGATGGTGACCC	TCGATGCAGGGCTTGACTG
Cyp27B1	GAGCAAACTCCAGGAAGCAG	TGAGGAATGATCAGGAGAGG
FGF23	CCATCTACAGTGCCCTGATG	GCTGAAGTGAAGCGATCC
GAPDH	CTGCACCACCAACTGCTTAGC	CATCCACAGTCTTCTGGGTG
hFGF23	GCTCTGGGTCTGTGCCTTGT	GTGATACTGAGGAGAGTG
klotho	CAATGGCTTTCCTCCTTTAC	TGCACATCCCACAGATAGAC
PTH	TGAGAGTCATTGTATGTAAAGATG	AGGTGTTTGCCCAGGTTG
slc34a1/NaPi2a	AGTCTCATTCGGATTTGGTGTCA	GCCGATGGCCTCTACCCT
slc34a3/NaPi2c	TAATCTTCGCAGTTCAGGTTGCT	CAGTGGAATTGGCAGTCTCAA
Tmem174	GCCACTTTGCTTTTCTCAGG	GGGACCCTCTCCTCGTTATC

### Protein sample purification and immunoblotting

BBMVs prepared using the Ca^2+^ precipitation method, cortical membrane, and whole homogenate were obtained from mouse kidneys and used for immunoblotting and immunoprecipitation analyses as described previously^35,45,48,49^. Protein samples were heated at 95 °C for 5 min in sample buffer in the presence of 2-mercaptoethanol and subjected to sodium dodecyl sulfate–polyacrylamide gel electrophoresis (SDS-PAGE). The separated proteins were transferred by electrophoresis to Immobilon-P polyvinylidene difluoride (Millipore, Billerica, MA, USA) and treated with diluted antibodies. Signals were detected using Immobilon Western (Millipore)^35,45,48,49^.

### Immunofluorescence staining

Mouse kidneys were fixed with the 4% paraformaldehyde solution (pH 7.2), overnight at 4 °C, washed with PBS, cryoprotected with 10% and 20% sucrose at 4 °C, embedded in Tissue-Tek O.C.T. Compound (Sakura Finetek Japan Co. Ltd., Tokyo, Japan), and frozen in hexane at − 80 °C. Frozen sections (5 μm thick) were collected onto MAS-coated slides (Matsunami Glass IND, Ltd., Osaka, Japan) and air-dried^44^. For immunofluorescence microscopy, serial frozen sections were incubated with primary antibodies overnight at 4 °C. Alexa Fluor 488 anti-rabbit (Molecular Probes, Eugene, OR, USA) and Alexa Fluor 568 anti-mouse (Molecular Probes) were used as secondary antibodies for 60 min at room temperature^44,45,47–49^. Thereafter, the sections were mounted with DAPI Fluoromount-G (SouthernBiotech, Birmingham, UK). Images were taken with an A1R confocal laser scanning microscope system (Nikon, Tokyo, Japan).

### Immunoprecipitation

Renal BBMVs of mice were lysed for 30 min at 4 °C in TNE lysis buffer (20 mM Tris–HCl, 150 mM NaCl, 1 mM EDTA, 1 mM EGTA, 1% TritonX-100, pH7.5), centrifuged for at 12,000×*g*, 10 min at 4 °C, and then supernatants were collected for immunoprecipitation. Immunoprecipitation samples were adjusted to 200 μg proteins/ ml in tubes, and anti-NHERF1 (LS-C46891, Lifespan Biosciences, Inc., Seattle, WA, USA) or anti- Tmem174 antibodies were added to tubes and rotated at 4 °C overnight. Next, protein A agarose beads (Santa Cruz Biotechnology, Inc., Dallas, TX, USA) were added to the tubes and rotated at 4 °C for 1 h. Protein A agarose beads were centrifuged at 3000×*g* for 1 min at 4 °C and washed with TNE lysis buffer 4 times before removing the supernatant and eluting in SDS sample buffer. Loading samples were heated at 95 °C for 5 min and then analyzed by SDS-PAGE using antibodies against NHERF1, Tmem174, and NaPi2a.

### Antibodies

Rabbit anti-NaPi2a and NaPi2c polyclonal antibodies were generated as described previously and used for immunoblotting and immunohistochemistry^7,44,47,48^. Rabbit anti- Tmem174 polyclonal antibodies were generated against mouse Tmem174 C-terminal fragments in rabbits as described previously^50^. Briefly, purified GST- Tmem174 C-terminal (residues 90-243) fusion proteins were used as antigens in rabbits. The purified IgG fractions were obtained from serums of the immunized animals. Mouse anti-actin monoclonal antibodies (Millipore) were used as an internal control. Horseradish peroxidase-conjugated anti-rabbit or anti-mouse IgG was utilized as the secondary antibody (Jackson ImmunoResearch Laboratories, Inc, West Grove, PA, USA). The diluted antibodies for immunoblotting were as follows: anti-NaPi2a (1:15,000), anti-NaPi2c (1:1500), anti-Tmem174 (1:1000), and anti-actin (1:10,000). The diluted antibodies for immunofluorescence staining were as follows: anti-NaPi2a (1:500), anti-NaPi2c (1:1000), anti-Tmem174 (1:200), anti-villin (Millipore; 1:300), and anti-NHERF1 (Lifespan Biosciences; 1:1000).

### Bone analysis

Histochemical analysis was performed as described previously^35,51^. The femora of all the groups were fixed with 4% paraformaldehyde overnight at 4 °C, decalcified for 4 weeks with 10% EDTA, and then embedded into paraffin for immunohistochemical examinations. For von Kossa staining, tibiae were immersed in a mixture containing 2% paraformaldehyde and 2.5% glutaraldehyde diluted in a 0.067 M cacodylate buffer (pH 7.4) and post-fixed with 1% osmium tetroxide in a 0.067 M cacodylate buffer for 4 h at 4 °C. After post-fixation, the tibiae were embedded in epoxy resin (Epon 812, TAAB Laboratories Equipment Ltd., Berkshire, UK) and sliced to semithin sections with 500 nm thickness using an ultramicrotome (Sorvall MT-5000; Ivan Sorvall, Inc., Norwalk, CT). Epoxy resin slides from undecalcified specimens were embedded in an aqueous solution of silver nitrate until a dark brown/black staining of the bone tissue was discernible under light microscopy.

Immunohistochemical analyses of mouse bone sections were performed as described previously. Briefly, the sections were immersed into 0.3% H_2_O_2_ in methanol for 30 min to block endogenous peroxidase. To reduce nonspecific binding, 1% bovine serum albumin (BSA; Serologicals Proteins Inc., Kankakee, IL, USA) in PBS (1% BSA-PBS) was applied to the sections for 20 min. The sections were then incubated with rabbit polyclonal antisera against tissue-nonspecific ALP diluted at 1:200^52^. A general method for rapid purification of soluble versions of glycosylphosphatidylinositol-anchored proteins expressed in insect cells: an application for human tissue-nonspecific alkaline phosphatase or rabbit polyclonal anti-dentin matrix protein-1 (Takara Bio) diluted at 1:200 with 1% BSA-PBS at room temperature for 2 h. The treated sections were incubated with horseradish peroxidase-conjugated anti-rabbit IgG antibody (Chemicon International, Temecula, CA, USA) for l h, and the immunoreactivities were visualized by using diaminobenzidine tetrahydrochloride as a substrate. For double detection of ALP and tartrate-resistant acid phosphatase, the sections immunodetected for ALP were incubated in a mixture of 8 mg of naphthol AS-BI phosphate (Sigma-Aldrich), 70 mg of red violet LB salt (Sigma-Aldrich), and 50 mM L ( +) tartaric acid (0.76 g; Nacalai Tesque, Kyoto, Japan) diluted in 60 ml of a 0.1 M sodium acetate buffer (pH 5.0) for 20 min at room temperature. Methyl green was used for counterstaining in all sections.

### Statistical analysis

Data are expressed as means ± SE. Differences among multiple groups were analyzed by analysis of variance followed by the Scheffe test. Differences between 2 experimental groups were established by analysis of variance followed by Student’s *t* test. A *P* value of less than 0.05 was considered significant.

## Supplementary Information

Supplementary Information.
