# Supplementary material for: Tmem174, a regulator of phosphate transporter prevents hyperphosphatemia
Source: Sci Rep. 2022 Apr 15;12:6353. doi: 10.1038/s41598-022-10409-3 (PMC9012787; doi:10.1038/s41598-022-10409-3)
Supplement: Supplementary file 1 — Supplementary Information. [file 41598_2022_10409_MOESM1_ESM.pdf]

Supplementary material

Tmem174, a regulator of phosphate transporter prevents hyperphosphatemia

Sumire Sasaki[1], Yuji Shiozaki[1], Ai Hanazaki[1], Megumi Koike[1], Kazuya Tanifuji[1], Minori Uga[1], Kota Kawahara[1], Ichiro Kaneko[1], Yasuharu Kawamoto[2], Pattama Wiriyasermkul[3], Tomoka Hasegawa[4], Norio Amizuka[4], Ken-ichi Miyamoto[1, 5], Shushi Nagamori[3*], Yoshikatsu Kanai[2*], Hiroko Segawa[1*]

***Corresponding authors:** Shushi Nagamori[3*], Yoshikatsu Kanai[2*], Hiroko Segawa[1*]

[1]Department of Applied Nutrition, Institute of Biomedical Sciences, Tokushima University Graduate School, Tokushima, Japan

[2]Department of Bio-system Pharmacology, Graduate School of Medicine, Osaka University, Osaka, Japan

[3]Department of Laboratory Medicine, The Jikei University School of Medicine, Tokyo, Japan

Supplementary information

Materials and Methods

Micro-CT analysis

Micro-CT images of the femora were obtained from $Tmem174^{+/+}$ and $Tmem174^{-/-}$ mice with a micro-CT unit (tube voltage 90 kV, CosmoScan FX, Rigaku Corporation, Tokyo, Japan). CT analyzer software (CosmoScan Viewer, Rigaku Corporation, Tokyo, Japan) was used for image reconstruction per the guidelines described by Bouxsein *et al.*[1]. For analysis of the bone mineral density (BMD, mg/mm^3), the regions of interest were defined as the articular surface of the distal femoral epiphysis and a diaphyseal area 8 mm away from the articular surface with inclusion of the cortical plates. The BMD of the femora of the $Tmem174^{+/+}$ and $Tmem174^{-/-}$ mice was analyzed using BMD Analysis software (Rigaku Corporation).

Bone histomorphometry analysis

Bone volume/tissue volume (BV/TV) and trabecular thickness (Tb.Th) were assessed in a region 900 μm x 1000 μm below the growth plate in the femoral metaphysis, excluding the cortical bone. Cortical thickness (Ct. Th) was measured in a region 2000 μm proximal to the distal growth plate in the femoral metaphysis. For analysis of the width of the growth plate, we used the growth plate of the distal femoral metaphysis. Whenever possible, the abbreviations used and calculations performed followed the recommendations of the ASBMR Histomorphometry Nomenclature Committee[2].

1. Bouxsein ML, Boyd SK, Christiansen BA*, et al.* Guidelines for assessment of bone microstructure in rodents using micro-computed tomography. *J Bone Miner Res* 2010; **25:** 1468-1486.

2. Parfitt AM, Drezner MK, Glorieux FH*, et al.* Bone histomorphometry: standardization of nomenclature, symbols, and units. Report of the ASBMR Histomorphometry Nomenclature Committee. *J Bone Miner Res* 1987; **2:** 595-610.

Supplemental Table 1 Co-expressed gene with slc34a1

RANK	Gene	Function	Entrez Gene ID	mmu-u.2 MR	mmu-m.4 MR	mmu-r.4 MR
0	Slc34a1	solute carrier family 34 (sodium phosphate): member 1	20505	0	0	0
1	Kap	kidney androgen regulated protein	16483	107.54	60.89	132.84
2	Tmem174	transmembrane protein 174	68344	158.16	61.63	283.01
3	Umod	uromodulin	22242	594.35	648.87	380.28
4	Pfn3	profilin 3	75477	595.91	5019.3	39.42
5	Defb29	defensin beta 29	75400	635.87	568.95	497.27
6	Slc12a3	solute carrier family 12: member 3	20497	635.95	489.27	578.41
7	Guca2b	guanylate cyclase activator 2b (retina)	14916	671.22	705.99	445.9
8	Cyp2j11	cytochrome P450: family 2: subfamily j: polypeptide 11	100066	705.29	904.22	382.82
9	Slc22a12	solute carrier family 22 (organic anion/cation transporter): member 12	20521	722.56	227.59	1560.19
10	Kcnj1	potassium inwardly-rectifying channel: subfamily J: member 1	56379	773.49	341	1212.5
11	Bsnd	barttin CLCNK type accessory beta subunit	140475	823.99	335.37	1392.72
12	Tmem52b	transmembrane protein 52B	330428	852.92	1258.75	399.53
13	Asb9	ankyrin repeat and SOCS box-containing 9	69299	857.22	311.74	1610.21
14	Slc5a10	solute carrier family 5 (sodium/glucose cotransporter): member 10	109342	871.48	310.09	1670.11
15	Mfsd4b5	major facilitator superfamily domain containing 4B5	215928	873.73	385.07	1367.78
16	Akr1c21	aldo-keto reductase family 1: member C21	77337	886.03	312.82	1709.65
17	Miox	myo-inositol oxygenase	56727	1171.62	306.17	2933.94
18	Mep1a	meprin 1 alpha	17287	1183.51	924.35	1060.32
19	Serpina1f	serine (or cysteine) peptidase inhibitor: clade A: member 1F	68348	1279.55	1128.35	1015.26
20	BC038268	cDNA sequence BC038268	100038756	1320.19	582.57	

Supplemental Table 2 Co-expressed gene with slc34a3

RANK	Gene	Function	Entrez Gene ID	mmu-u.2 MR	mmu-m.4 MR	mmu-r.4 MR
0	Slc34a3	solute carrier family 34 (sodium phosphate): member 3	1	0	0	0
1	Slc5a11	solute carrier family 5 (sodium/glucose cotransporter): member 11	233836	804.13	286	1544.78
2	Clcnkb	chloride channel: voltage-sensitive Kb	56365	1066.11	153.95	4433.95
3	Nkx2-9	NK2 homeobox 9	18094	1182.93	1055.99	927.12
4	Dao	D-amino acid oxidase	13142	1326.48	1094.31	1125.28
5	Cyp2j11	cytochrome P450: family 2: subfamily j: polypeptide 11	100066	1587.03	979.45	1789.51
6	Kap	kidney androgen regulated protein	16483	1595.69	997.89	1776.49
7	Slc12a3	solute carrier family 12: member 3	20497	1613.82	1546.83	1176.57
8	Miox	myo-inositol oxygenase	56727	1657.05	664.67	2788.22
9	Aqp6	aquaporin 6	11831	1843.88	2214.76	1062.15
10	A930017K11Rik	RIKEN cDNA A930017K11 gene	100034748	1877.91		794.15
11	Prima1	proline rich membrane anchor 1	170952	1898.83	798.3	3048.04
12	Mep1b	meprin 1 beta	17288	2019.99	657.64	4032.44
13	Tmem174	transmembrane protein 174	68344	2068.14	2155.02	1382.27
14	Clcnka	chloride channel: voltage-sensitive Ka	12733	2094.74	1345.05	2270.18
15	Slc7a9	solute carrier family 7 (cationic amino acid transporter: y+ system): member 9	30962	2109.07	1846.57	1685.34
16	Nphs1	nephrosis 1: nephrin	54631	2162.82	1264.26	2561.16
17	Slc22a12	solute carrier family 22 (organic anion/cation transporter): member 12	20521	2178.43	1063.32	3044.96
18	Pga5	pepsinogen 5: group I	58803	2223.02	3012.04	1119.69
19	Serpina1f	serine (or cysteine) peptidase inhibitor: clade A: member 1F	68348	2260.84	1836.68	1947.56
20	Slc22a7	solute carrier family 22 (organic anion transporter): member 7	108114	2297.5	1230.79	2947.09

Supplemental Table 3 Plasma biochemistry parameters

	Tmem174$^{+/+}$ (n=5)	Tmem174$^{+/-}$ (n=5)	Tmem174$^{-/-}$ (n=5)
Na (mmol/L)	162.3±1.1	162.1±0.8	162.0±0.4
K (mmol/L)	4.15±0.33	4.88±0.39	5.37±0.48
Cl (mmol/L)	123.2±0.7	125.2±1.1	121.7±0.8
Fe (mg/dL)	150±14	143±15	132±10
Mg (mg/dL)	2.4±0.1	2.5±0.1	2.4±0.1
Total protein (g/dL)	4.01±0.16	4.31±0.19	4.37±0.13
Albumin (g /dL)	2.45±0.15	2.73±0.09	2.79±0.05
Total bilirubin (mg/dL)	0.04±0.01	0.03±0.01	0.05±0.03
Total bile Acid (mmol/L)	1.13±0.33	1.20±0.26	1.00±0.12
Aspartate aminotransferase (u/L)	43±7	62±11	45±4
Alanine aminotransferase (u/L)	30±7	52±13	29±4
Alkaline phosphatase (u/L)	155±9	136±9	142±12
creatinine (mg/dL)	0.22±0.007	0.23±0.004	0.23±0.003
urea nitrogen (mg/dL)	25.5±1.9	31.7±0.7	23.9±1.9
N-acetyl-β-D-glucosaminidase (u/L)	18.7±2.1	18.6±0.7	18.5±1.0
triglyceride (mg/dL)	36±8	71±27	61±20
Total-cholesterol (mg/dL)	76±6	84±6	80±10
LDL-cholesterol (mg/dL)	5±0.0	6±0.3	7±0.5
glucose (mg/dL)	277±16	278±39	238±24

Values are mean ± SE

Supplemental Figure S1

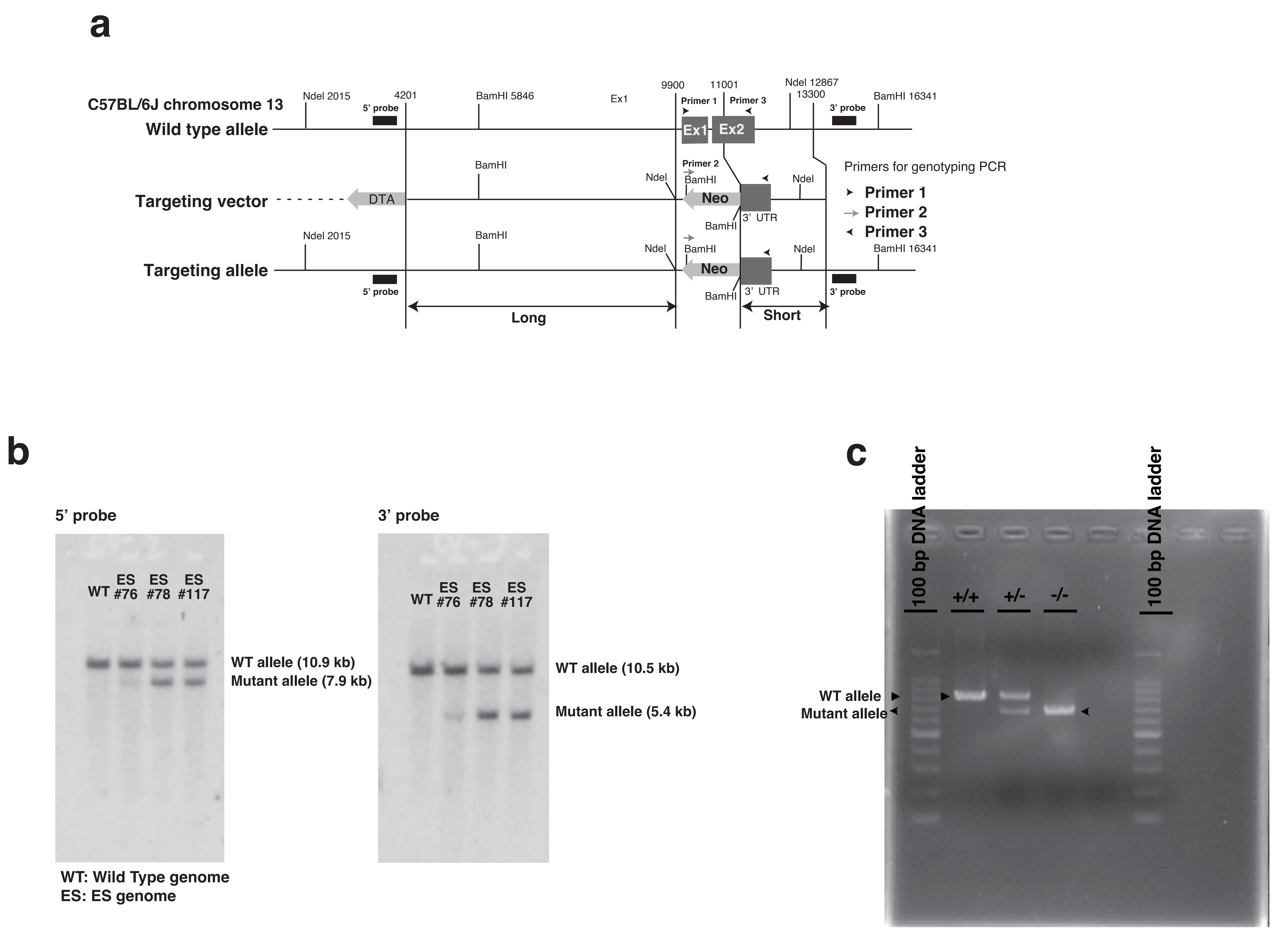

Supplemental Figure S1 Establishment of Tmem174-null mice

(a) Wild-type allele and targeted mutant allele. Allows meaning genotyping primers. (b) Southern blot analysis. Genomic DNA (5 μg) isolated from transfected ES clones were digested with NdeI and BamHI and hybridized to the 5' or 3' probes. (c) PCR analysis of mouse tail genomic DNA from Tmem174$^{+/+}$, Tmem174$^{+/-}$, and Tmem174$^{-/-}$ mice.

Supplemental Figure S2

a 8-week-old

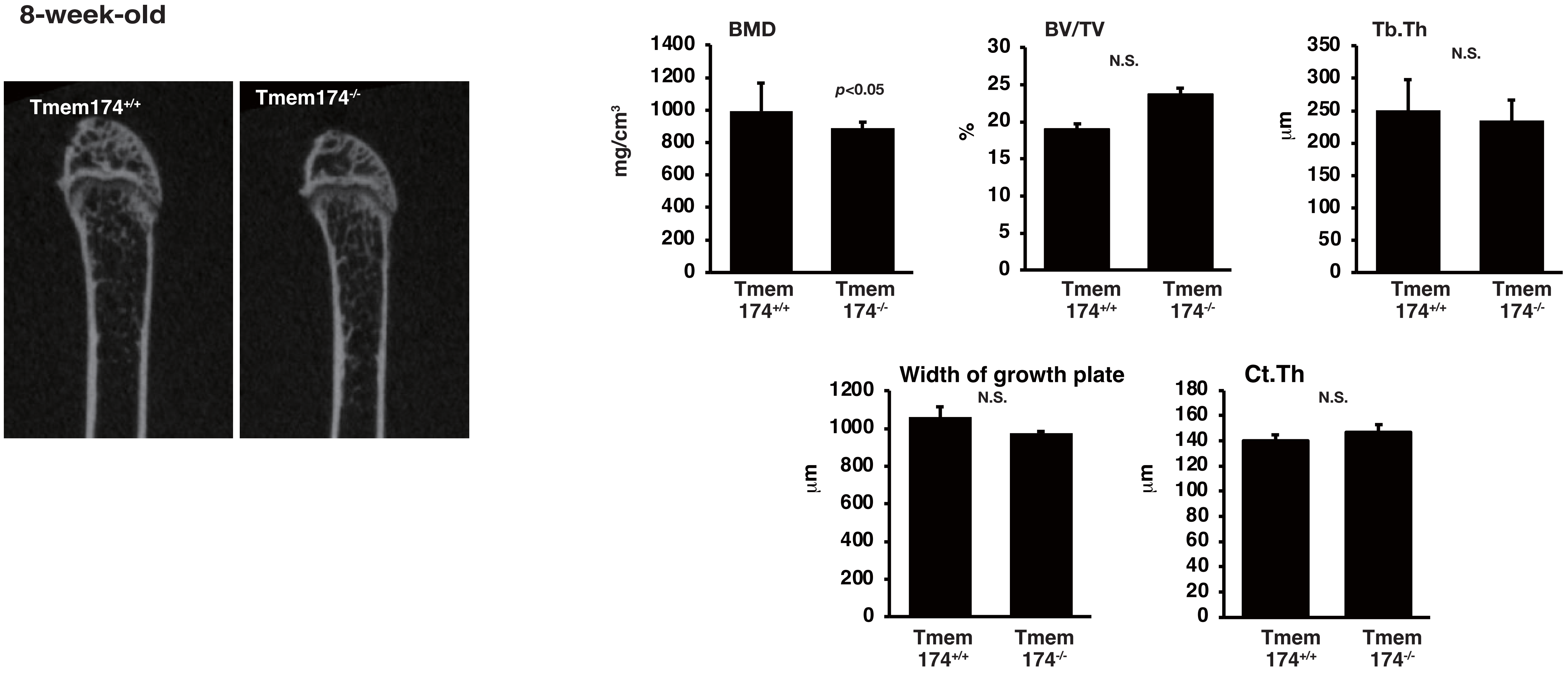

b 70-week-old

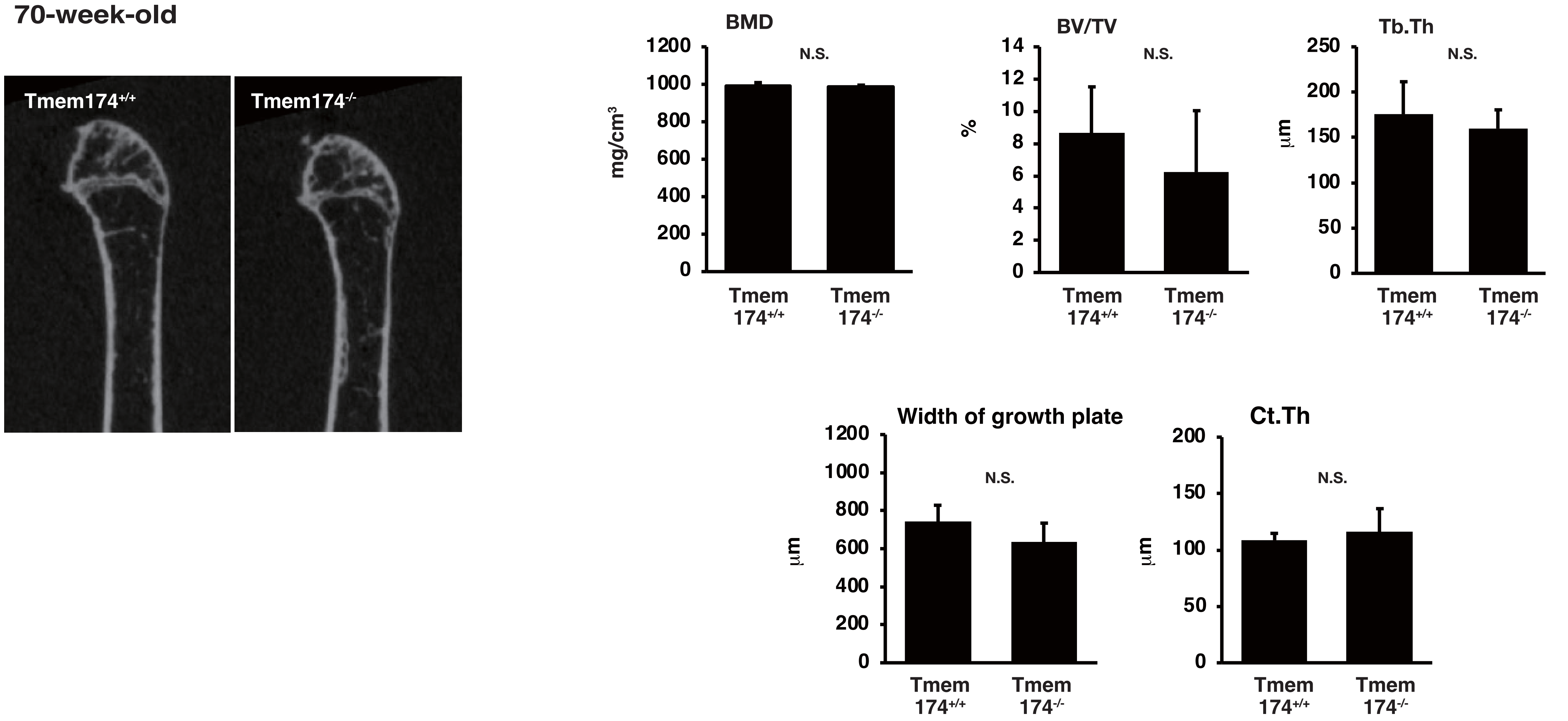

Supplemental Figure S2 Micro-CT analyses and total and trabecular bone parameters

Femurs in male Tmem174$^{+/+}$ and Tmem174$^{-/-}$ mice at 8 weeks and 70 weeks of age. Bone structure and bone mineral density (BMD) by Micro-CT analyses and bone histomorphometry analysis including bone volume/tissue volume (BV/TV), trabecular thickness (Tb.Th), cortical thickness (Ct. Th), and width of growth plate in 8 weeks (a) and 70 weeks (b) of age. Values are mean ± SE (n=3).

Supplemental Figure S3

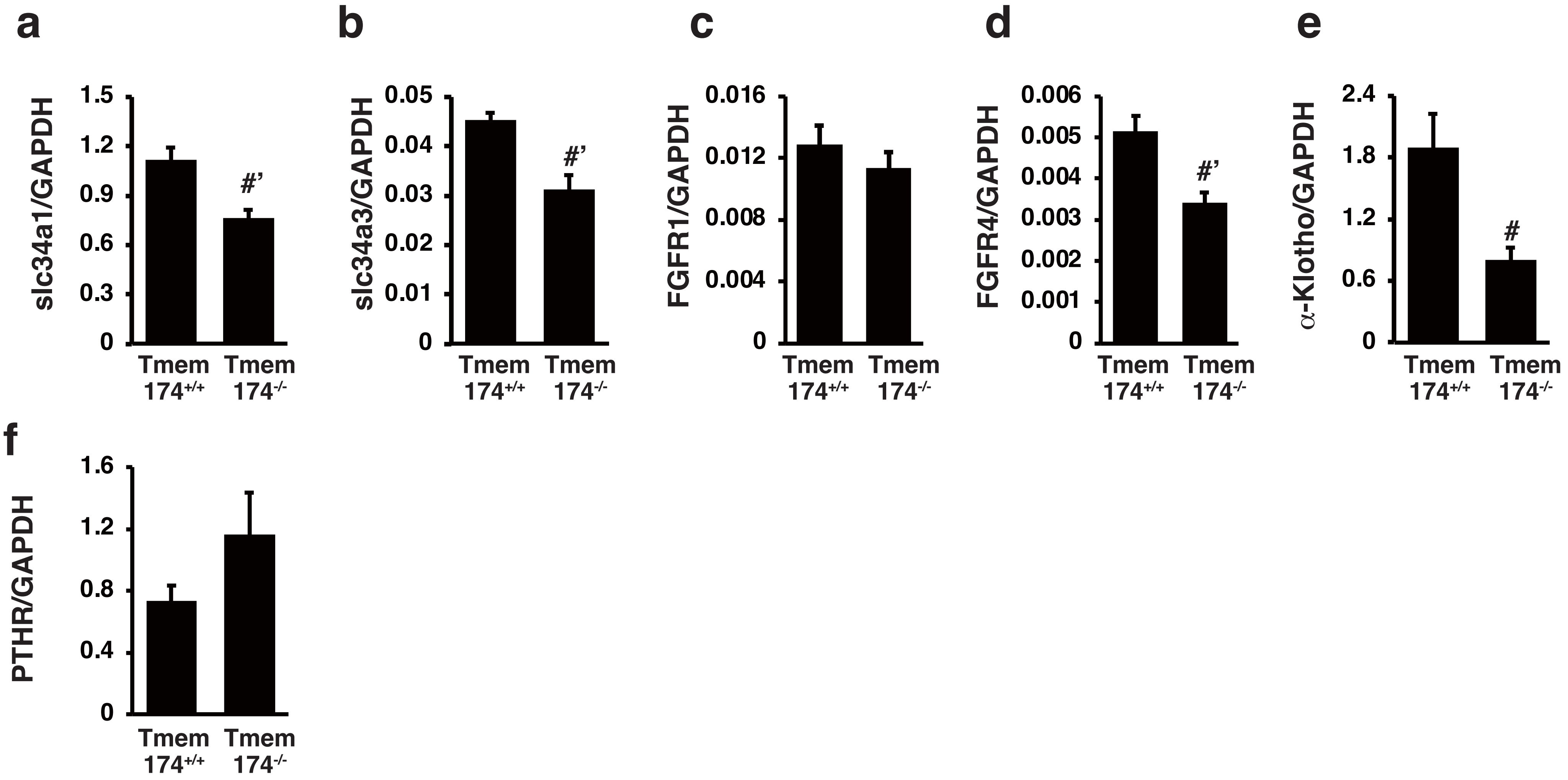

Supplemental Figure S3 Effects of Tmem174 deletion on mRNA of NaPi transporters and related genes

(a) slc34a1, (b) slc34a3, (c) FGFR1, (d) FGFR4, (e) α-Klotho, and (f) PTHR mRNA levels in the kidney by real-time PCR analysis. Male mice at 8 weeks of age (n=10-15) were used. GAPDH was used as an internal control. The relative intensity of transporter expression in $Tmem174^{+/+}$ mice was defined as 1.0. Values are mean ± SE. #$p<0.05$, #' $p<0.01$.

Supplemental Figure S4

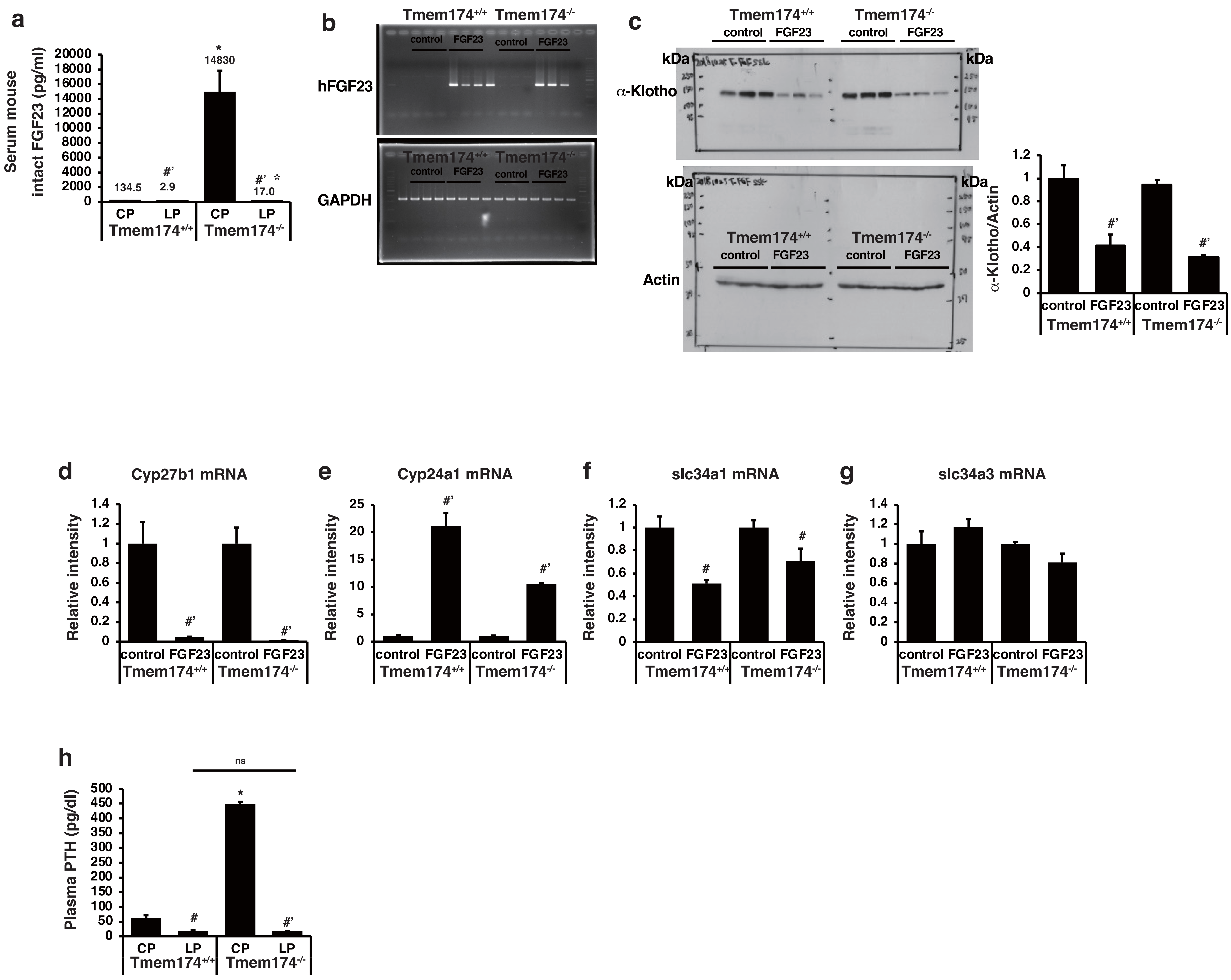

Supplemental Figure S4 Abnormal regulation of phosphaturic action on renal NaPi2a Pi transporter in Tmem174$^{-/-}$ mice.

(a) Serum intact FGF23. Values are mean ± SE. The average value is shown at the top of the column.
#' p<0.01 vs CP (same genotype), *p<0.01 vs Tmem174$^{+/+}$ mice (same diet). (b) RT-PCR of humanFGF23 mRNA in mouse liver. GAPDH was used as an internal control. (c) Western blotting analysis of α-Klotho. Each lane was loaded with 20 μg of a cortical membrane of the kidneys. Actin was used as an internal control. The blot was cut prior to hybridization with α-Klotho antibody.Values are mean ± SE. #' p<0.01 vs control (same genotype). Real-time PCR for Cyp27b1 (d), Cyp24a1 (e), slc34a1 (f), and slc34a3 (g) mRNA in mouse kidney. Values are mean ± SE. #p<0.05, #' p<0.01 vs control (same genotype).
(h) Plasma PTH. Values are mean ± SE. #' p<0.01 vs CP (same genotype), *p<0.01 vs Tmem174+/+ mice (same diet).
ns; not significant.

Supplemental Figure S5

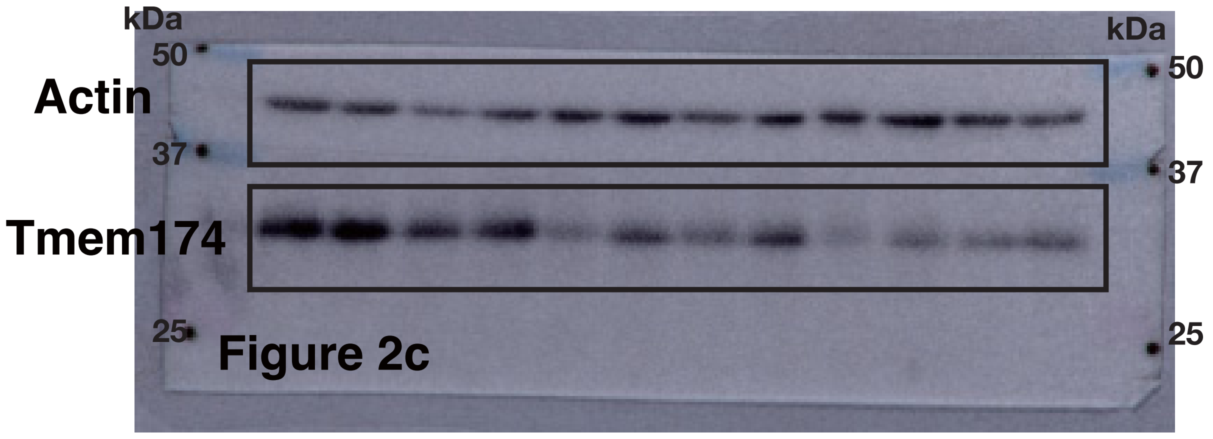

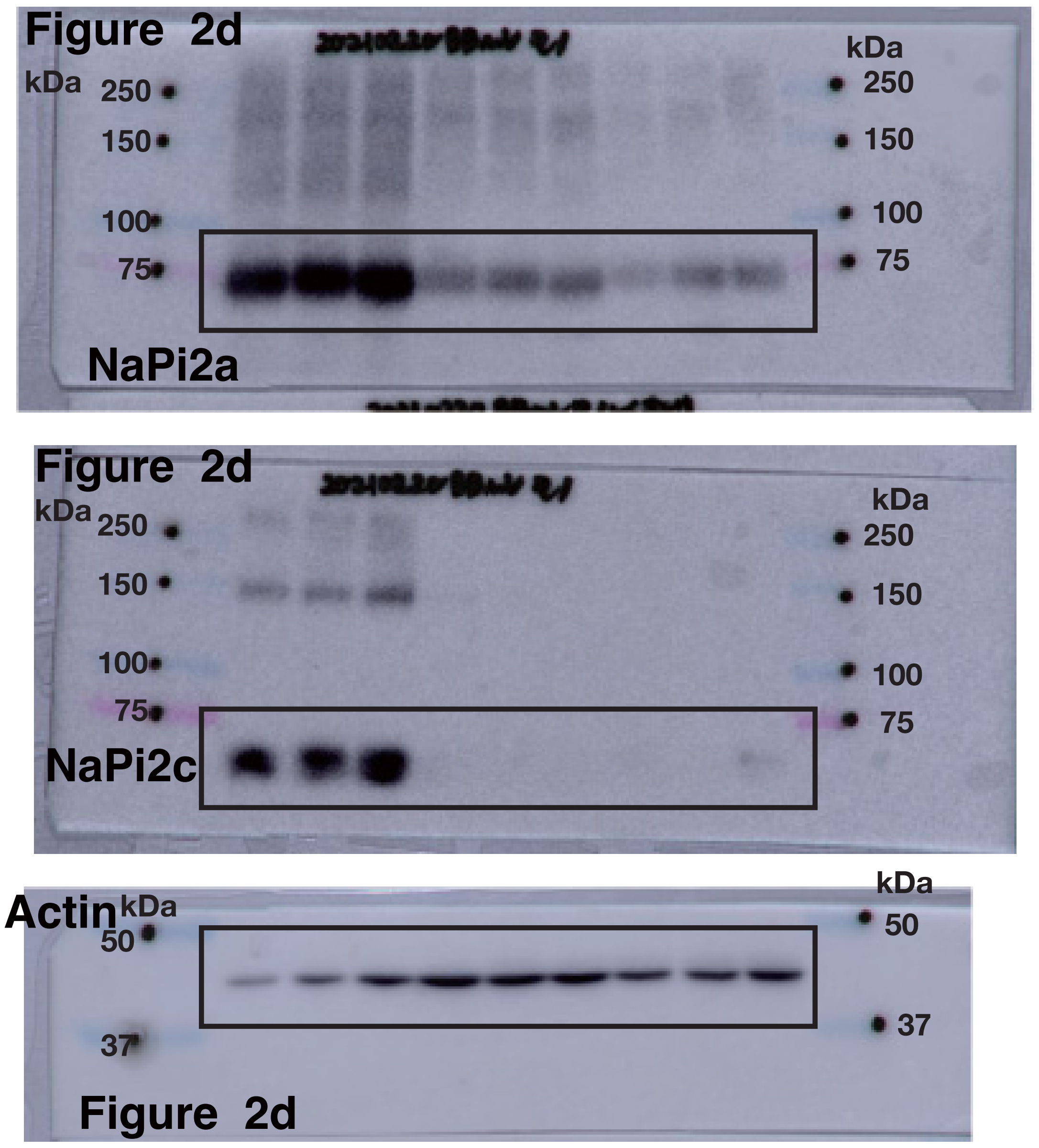

Figure 2e

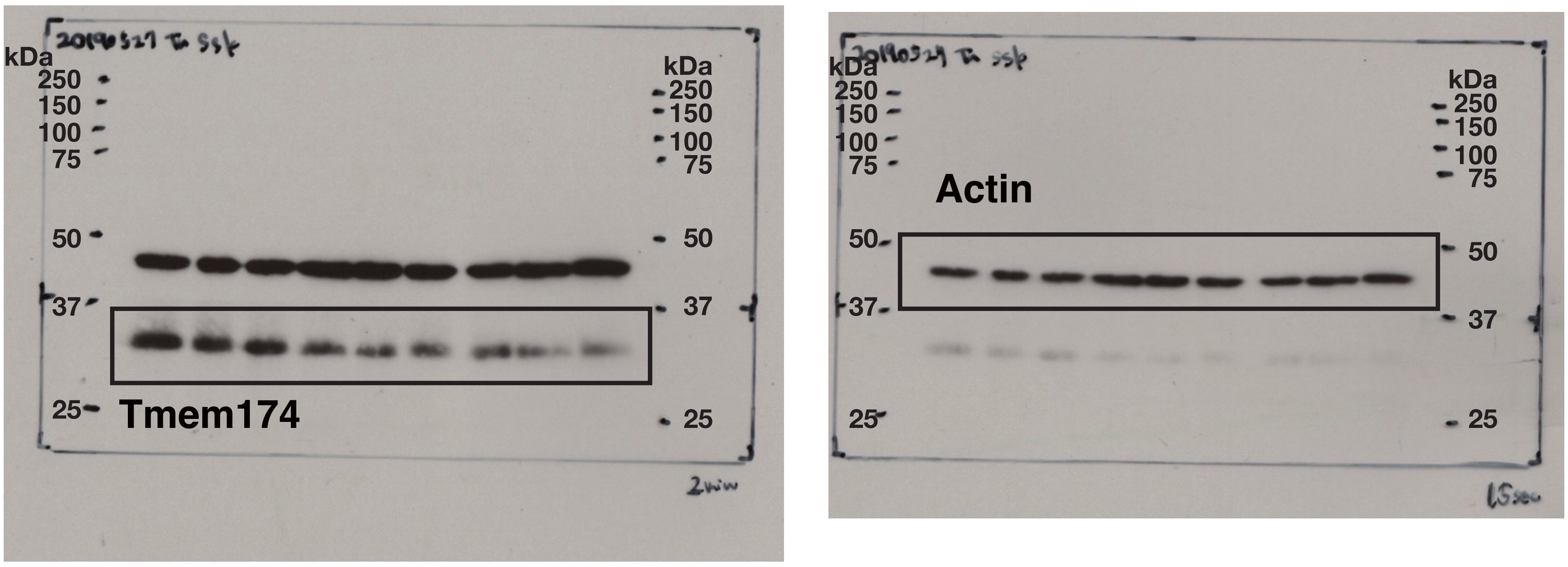

Supplementary Figure S5

Originary Western blotting images. Rectangles indicate the area shown in Figure 2c-2e.

Each blots were cut prior to hybridization with antibodies.

Supplemental Figure S6

Figure 3a

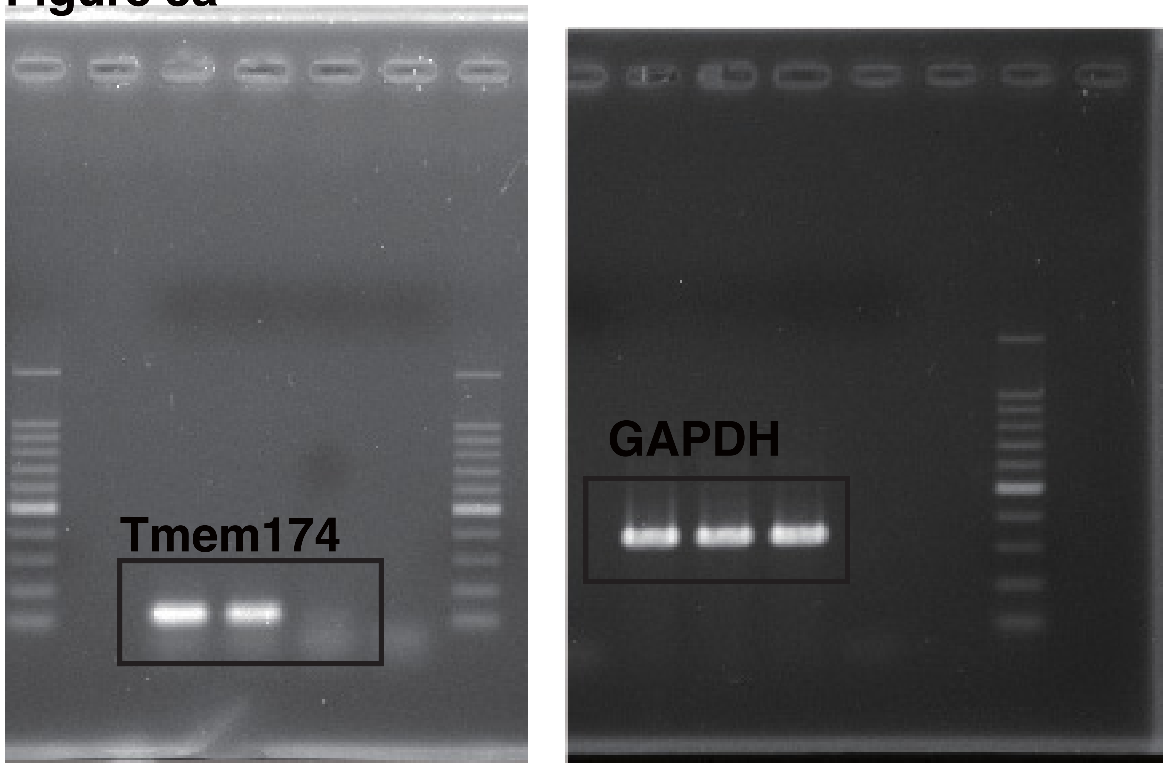

Figure 3b

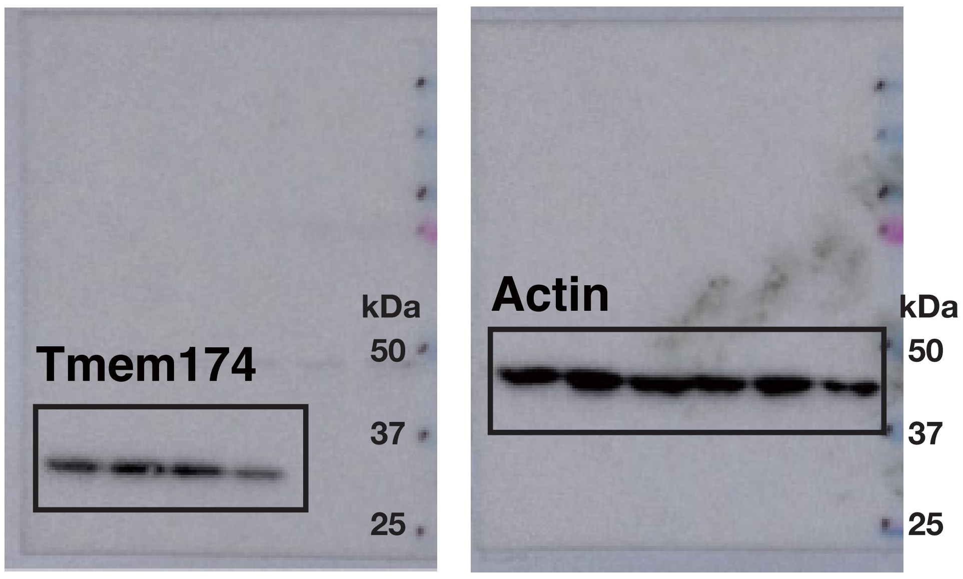

Supplementary Figure S6

Originary Western blotting images. Rectangles indicate the area shown in Figure 3a, 3b.

Supplemental Figure S7

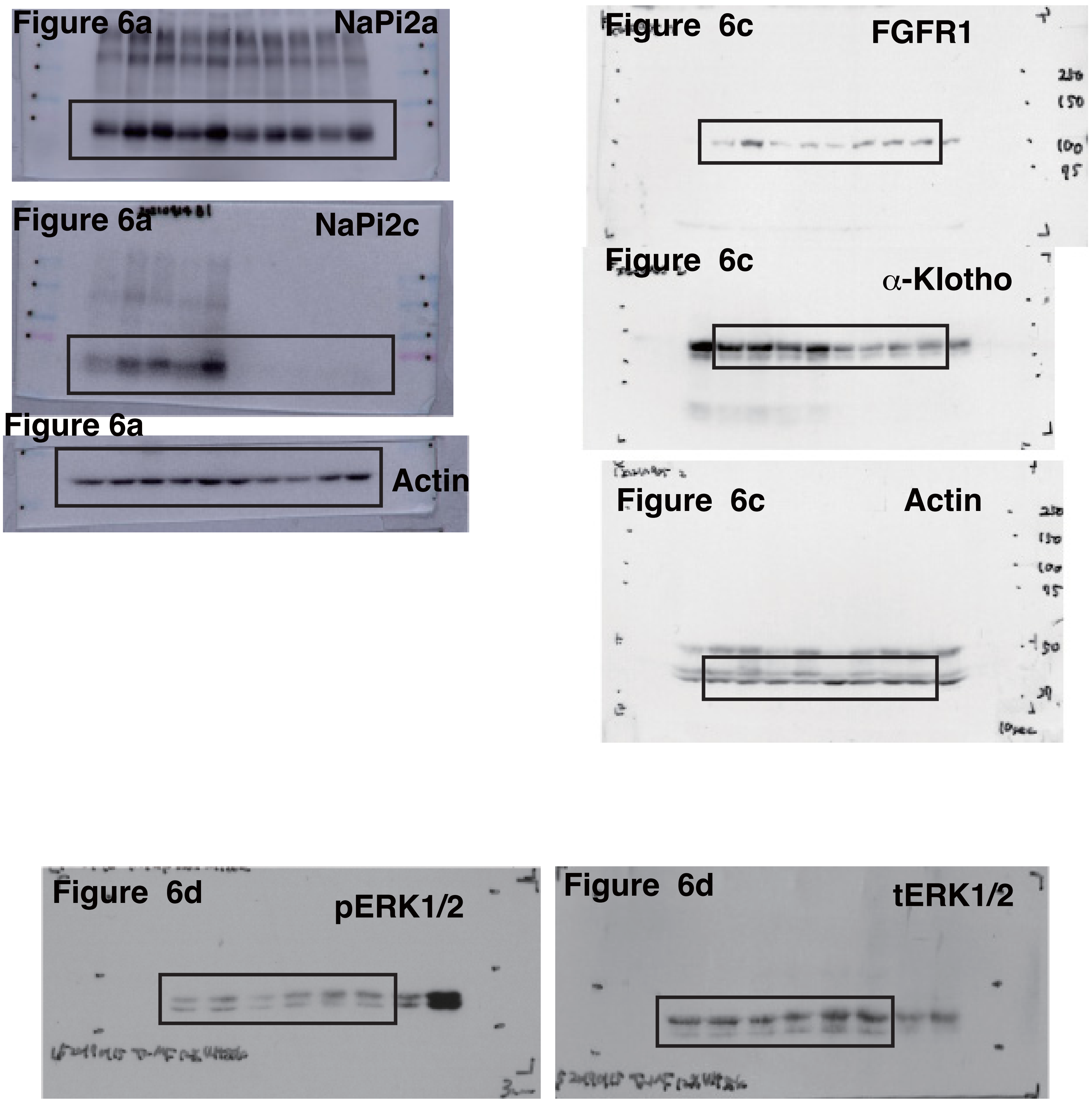

Supplementary Figure S7

Originary Western blotting images. Rectangles indicate the area shown in Figure 6a, 6c, 6d. Each blots were cut prior to hybridization with antibodies (Figs. 6a).

Supplemental Figure S8

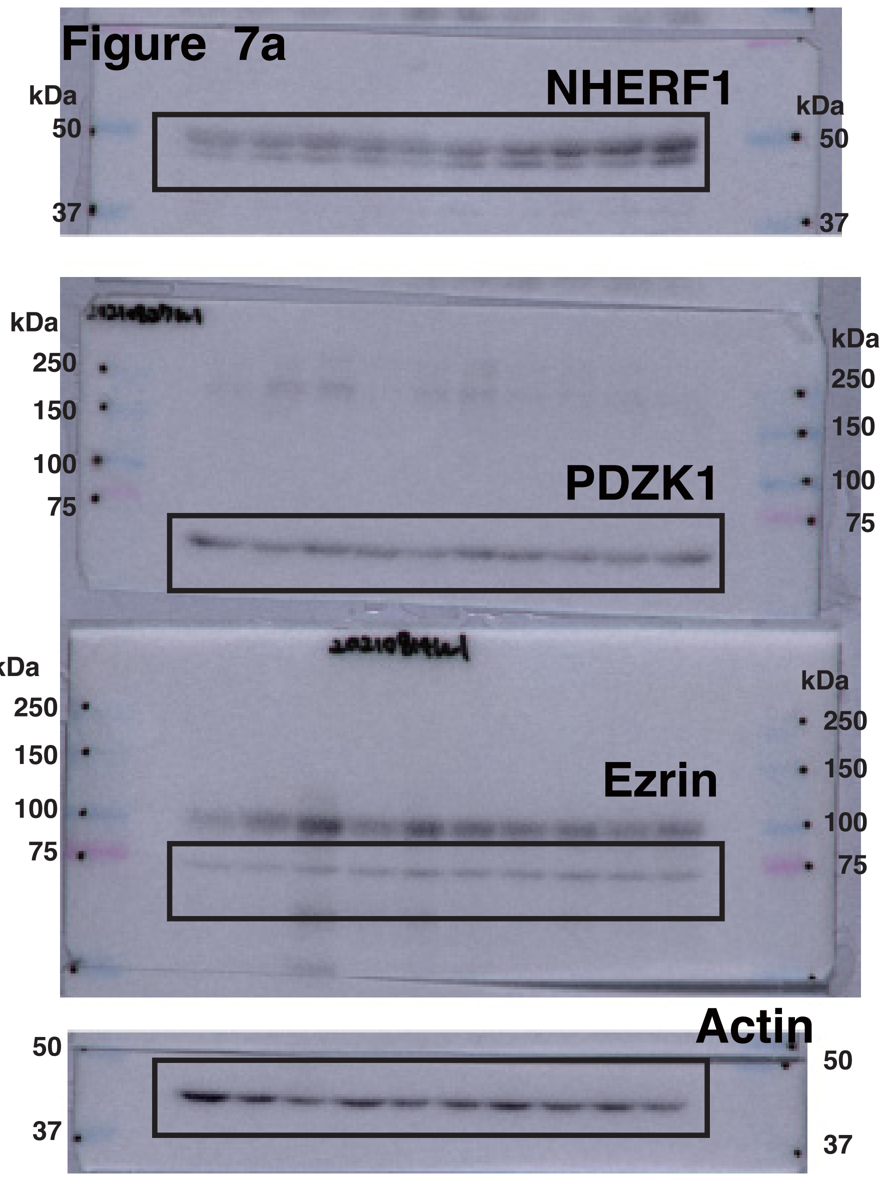

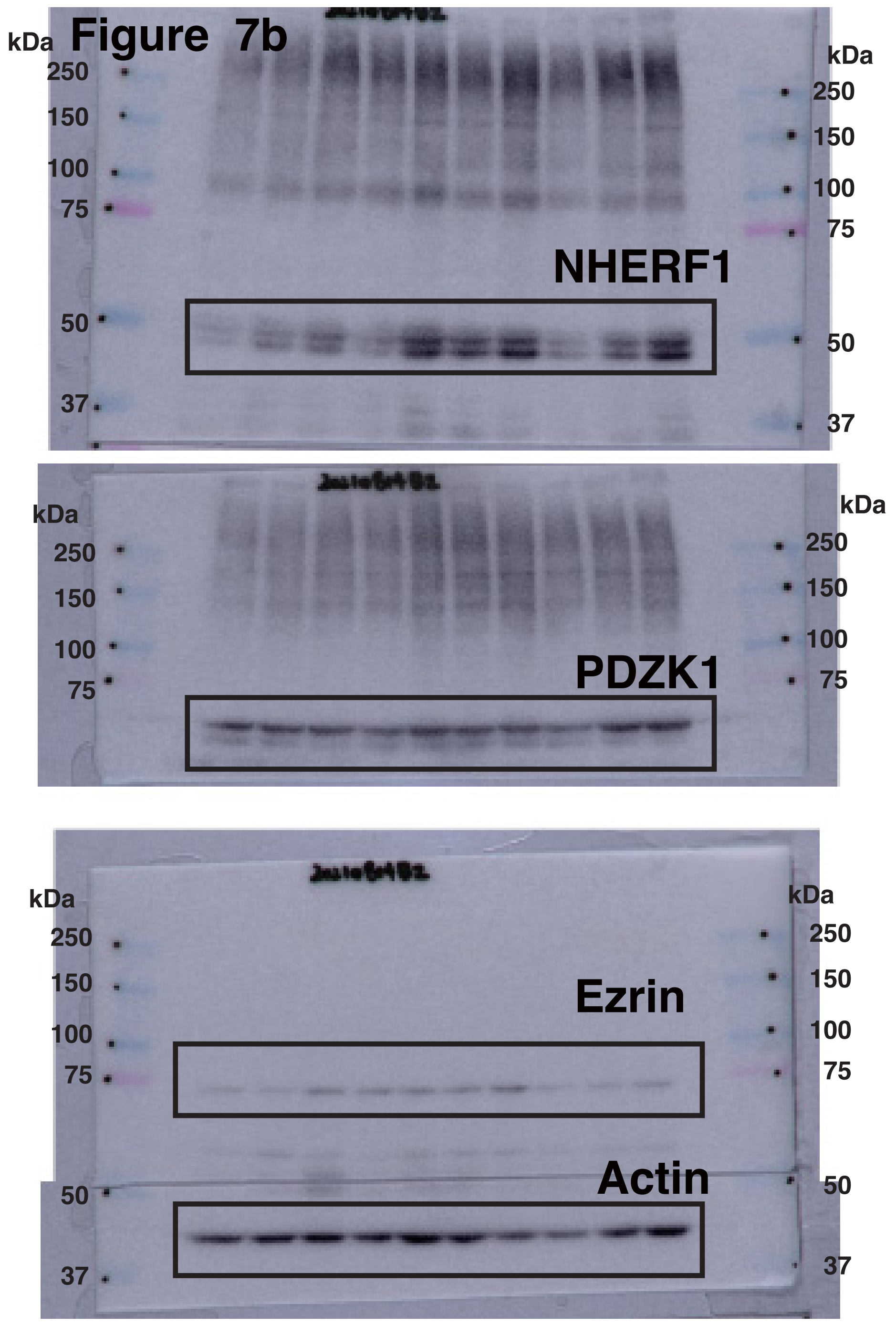

Figure 7c

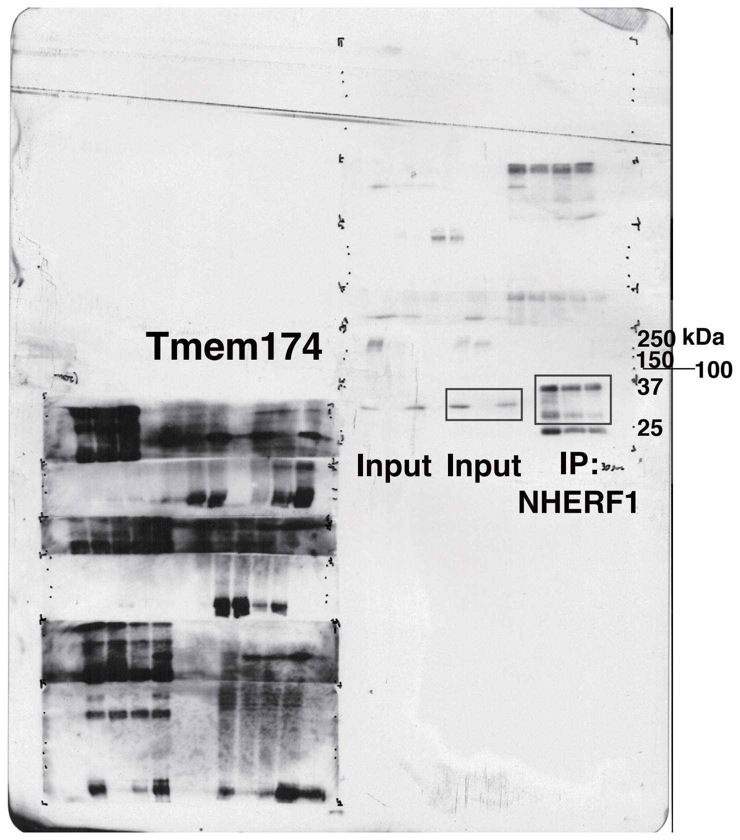

Figure 7c

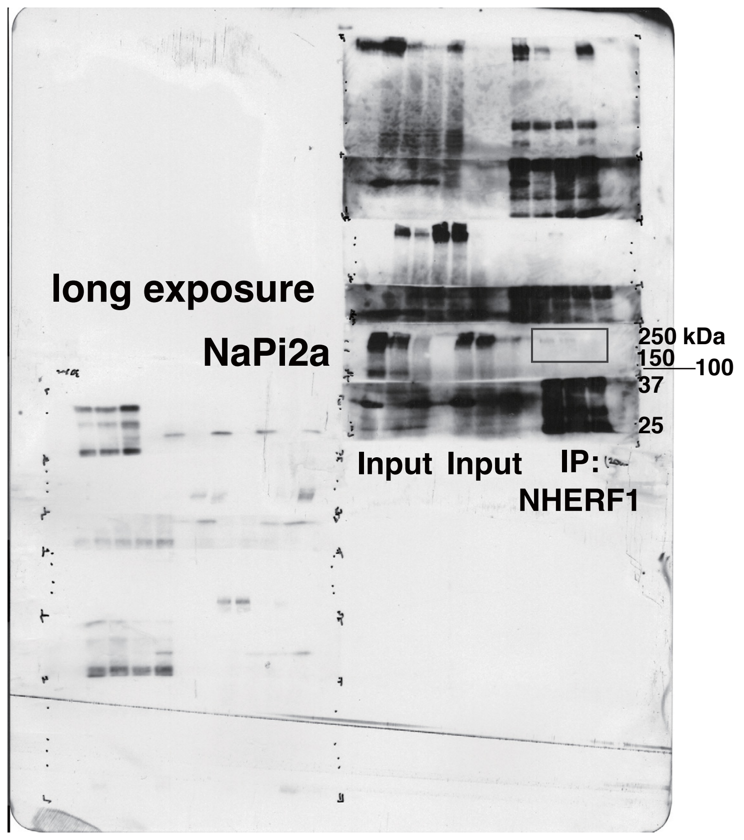

Figure 7c

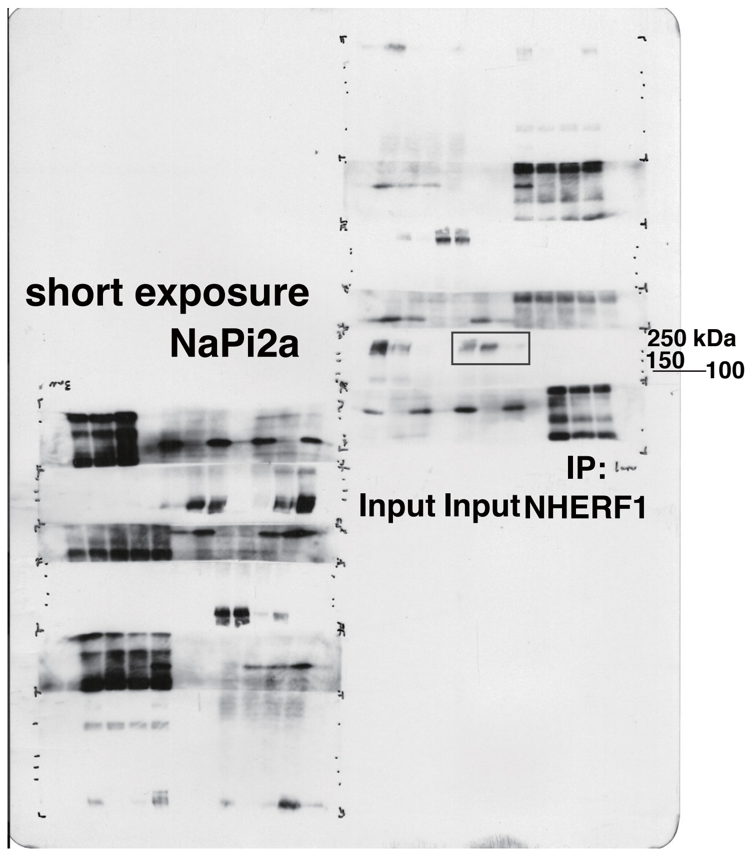

Figure 7c

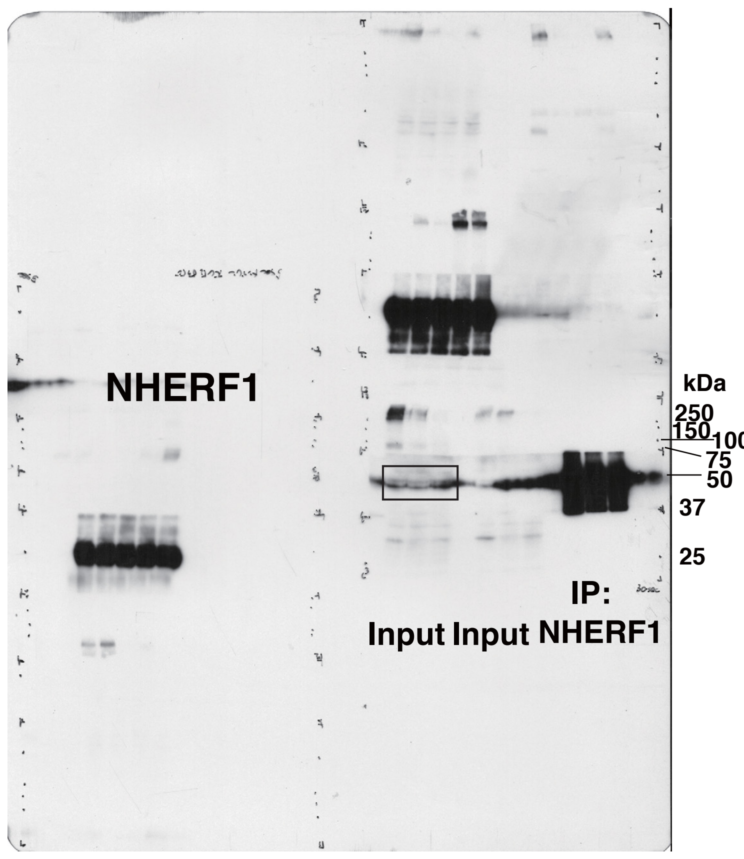

Figure 7d

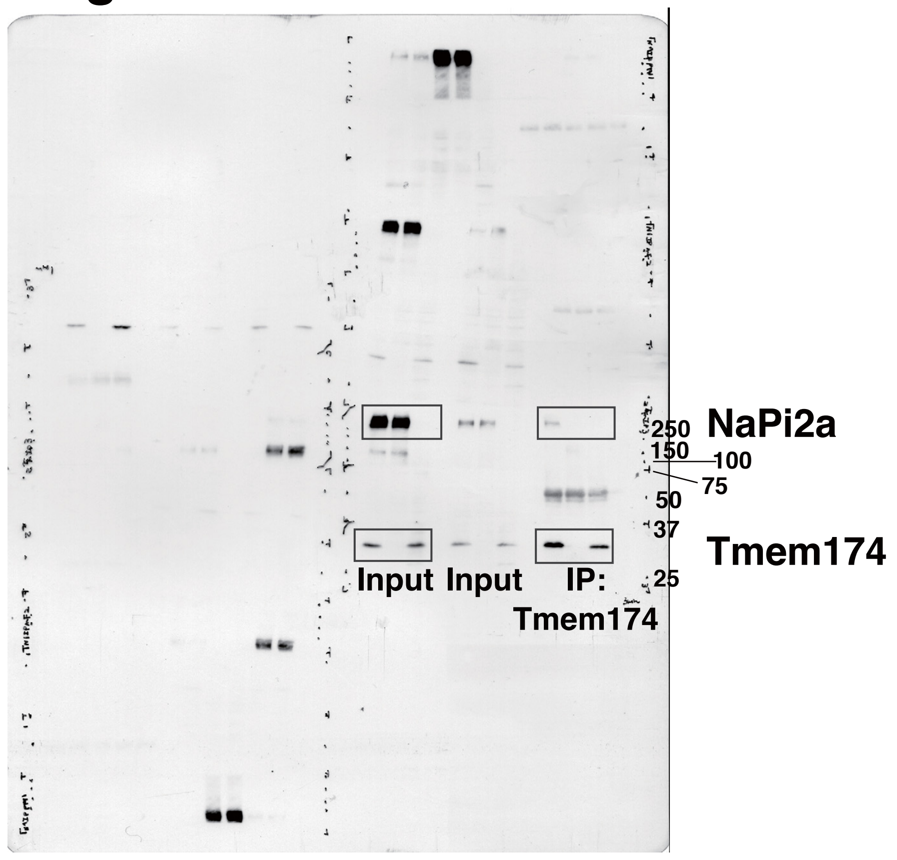

Supplementary Figure S8

Originary Western blotting images. Rectangles indicate the area shown in Figure 7a-7e.

Each blots were cut prior to hybridization with antibodies.

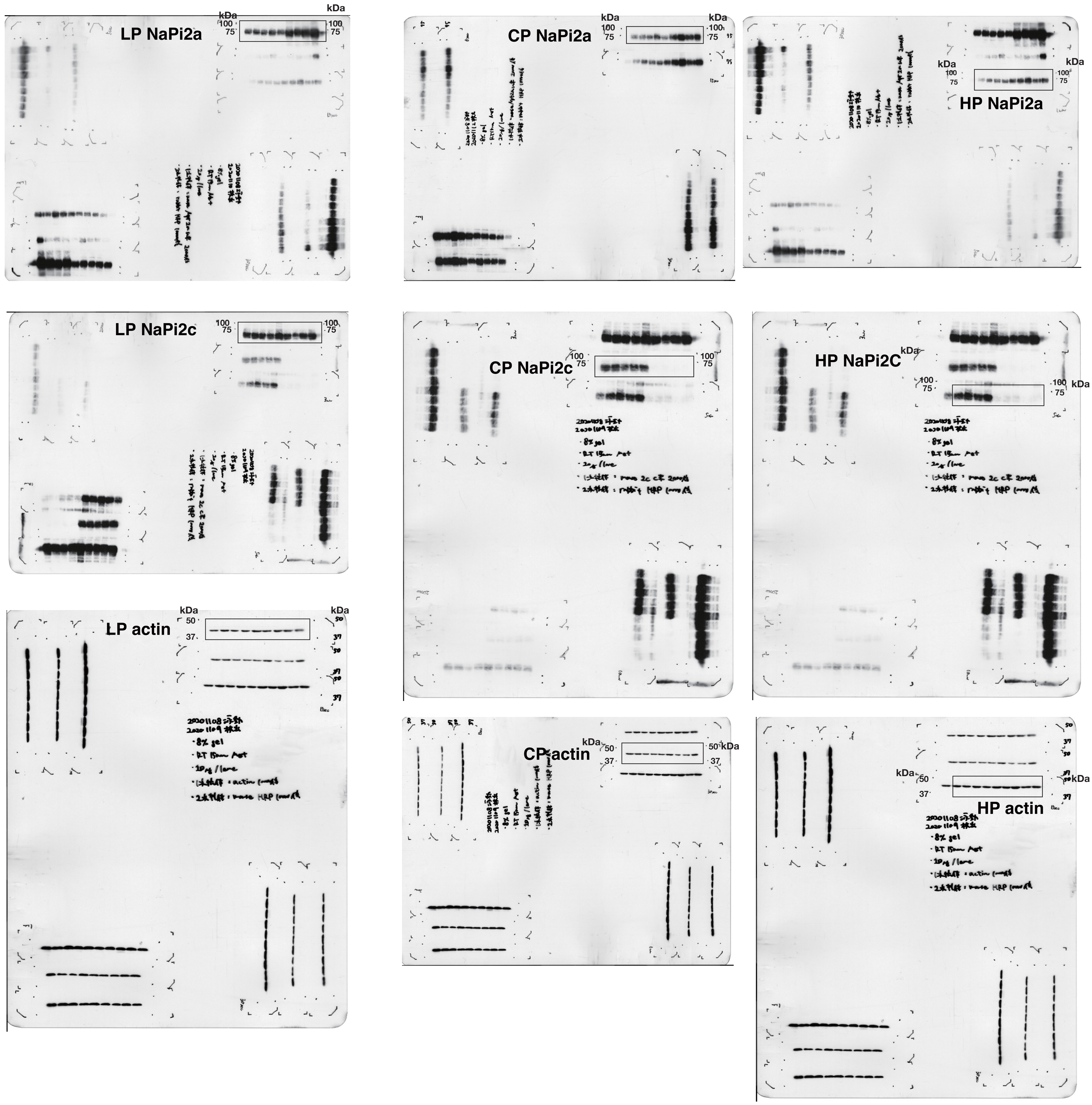

Supplementary Figure S9

Originary Western blotting images. Rectangles indicate the area shown in Figure 8c.

Each blots were cut prior to hybridization with antibodies.

Supplemental Figure S10

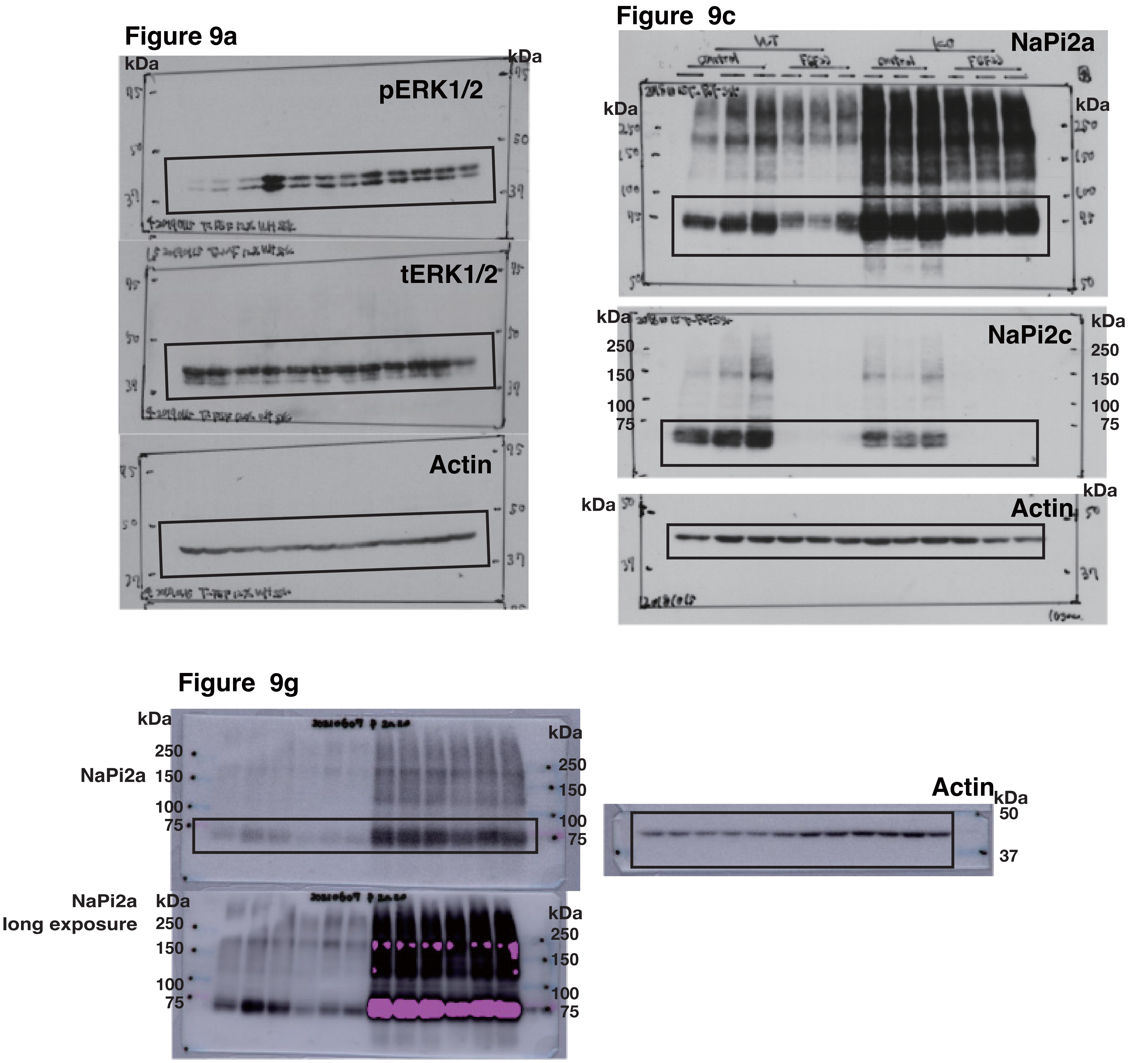

Supplementary Figure S10

Originary Western blotting images. Rectangles indicate the area shown in Figure 9a, 9c, 9g.

Each blots were cut prior to hybridization with antibodies.

Supplemental Figure S11

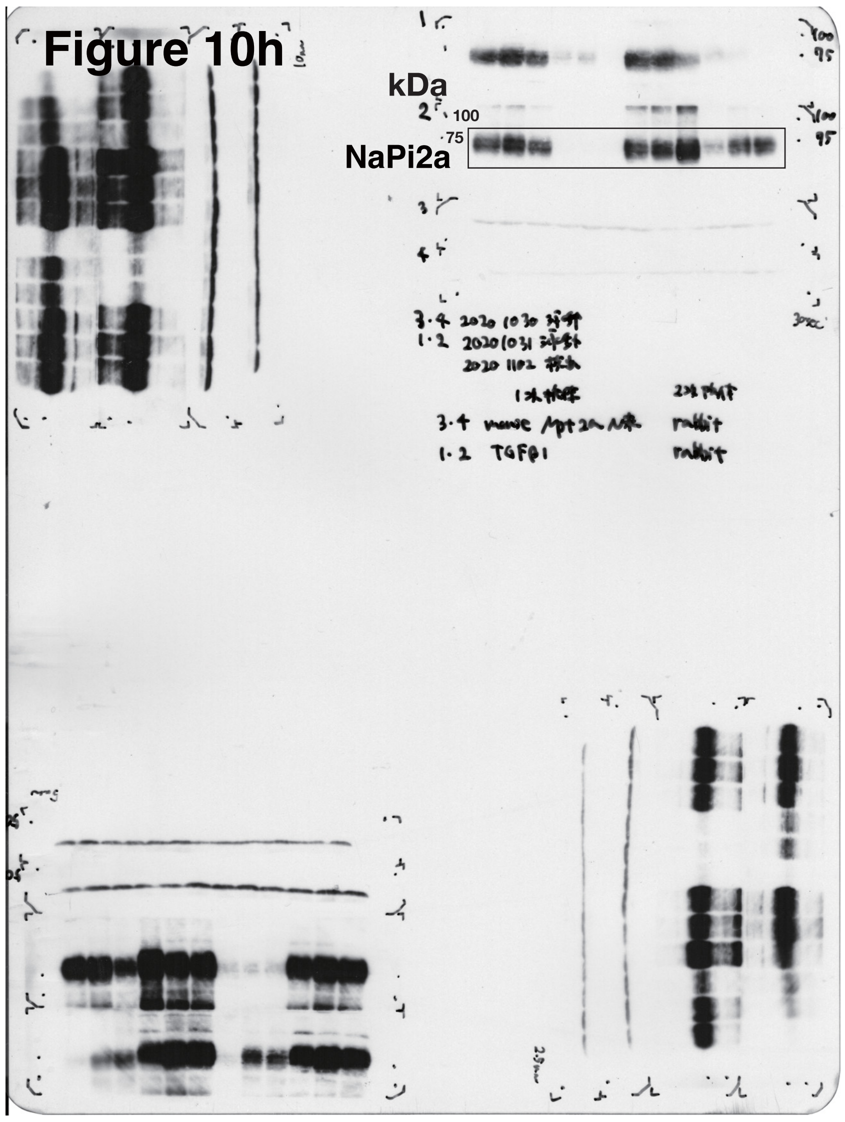

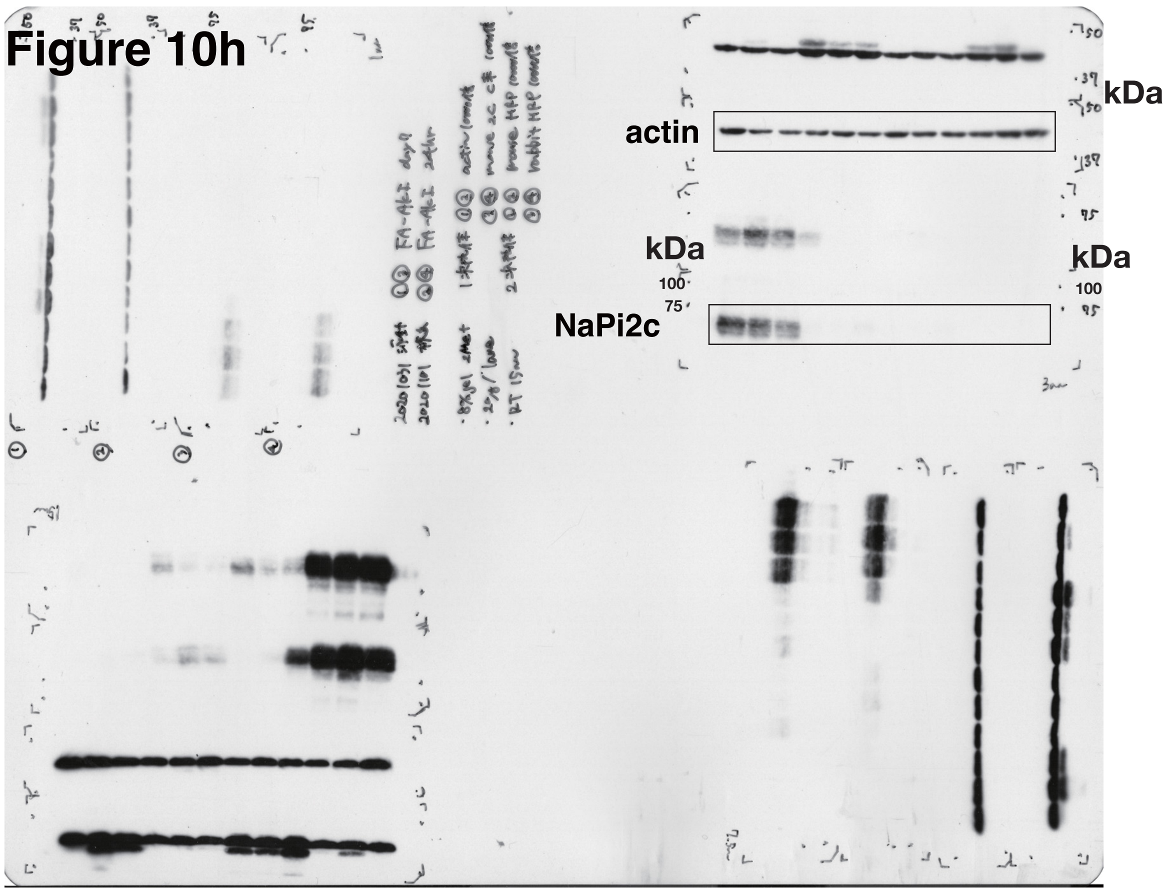

Supplementary Figure S11 Renal injury in Tmem174$^{-/-}$ mice

Originary Western blotting images. Rectangles indicate the area shown in Figure 10h.

Each blots were cut prior to hybridization with antibodies.
